# Stochastic Compartment Model with Mortality and Its Application to Epidemic Spreading in Complex Networks

**DOI:** 10.3390/e26050362

**Published:** 2024-04-25

**Authors:** Téo Granger, Thomas M. Michelitsch, Michael Bestehorn, Alejandro P. Riascos, Bernard A. Collet

**Affiliations:** 1 Sorbonne Université, Institut Jean le Rond d’Alembert, CNRS UMR 7190, 4 Place Jussieu, 75252 Paris, Cedex 05, Francebernard.collet@upmc.fr (B.A.C.); 2 Institut für Physik, Brandenburgische Technische Universität Cottbus-Senftenberg, Erich-Weinert-Straße 1, 03046 Cottbus, Germany; bestehorn@b-tu.de; 3 Departamento de Física, Universidad Nacional de Colombia, Bogotá, Colombia; alperezri@unal.edu.co

**Keywords:** epidemic spreading, compartment model with mortality, memory effects, random walks, random graphs

## Abstract

We study epidemic spreading in complex networks by a multiple random walker approach. Each walker performs an independent simple Markovian random walk on a complex undirected (ergodic) random graph where we focus on the Barabási–Albert (BA), Erdös–Rényi (ER), and Watts–Strogatz (WS) types. Both walkers and nodes can be either susceptible (S) or infected and infectious (I), representing their state of health. Susceptible nodes may be infected by visits of infected walkers, and susceptible walkers may be infected by visiting infected nodes. No direct transmission of the disease among walkers (or among nodes) is possible. This model mimics a large class of diseases such as Dengue and Malaria with the transmission of the disease via vectors (mosquitoes). Infected walkers may die during the time span of their infection, introducing an additional compartment D of dead walkers. Contrary to the walkers, there is no mortality of infected nodes. Infected nodes always recover from their infection after a random finite time span. This assumption is based on the observation that infectious vectors (mosquitoes) are not ill and do not die from the infection. The infectious time spans of nodes and walkers, and the survival times of infected walkers, are represented by independent random variables. We derive stochastic evolution equations for the mean-field compartmental populations with the mortality of walkers and delayed transitions among the compartments. From linear stability analysis, we derive the basic reproduction numbers $R_{M},R_{0}$ with and without mortality, respectively, and prove that $R_{M}<R_{0}$. For $R_{M},R_{0}>1$, the healthy state is unstable, whereas for zero mortality, a stable endemic equilibrium exists (independent of the initial conditions), which we obtained explicitly. We observed that the solutions of the random walk simulations in the considered networks agree well with the mean-field solutions for strongly connected graph topologies, whereas less well for weakly connected structures and for diseases with high mortality. Our model has applications beyond epidemic dynamics, for instance in the kinetics of chemical reactions, the propagation of contaminants, wood fires, and others.

## 1. Introduction

The sudden or recurrent emergence of epidemics has been an everlasting threat to humanity. Highly infectious and fatal diseases such as pestilence, typhus, cholera, and leprosy were among the main causes of death in medieval times in Europe and, until the 20th century, a major scourge of humanity [1]. This permanent challenge has naturally driven interest in protective measures and predictive models.

The systematic mathematical study of epidemic spreading began only a century ago with the seminal work of Kermack and McKendrick [2]. They were the first to introduce what we call currently a “compartment model”. In their so-called SIR model, the individuals are categorized into the compartments susceptible (S), infected (I), and recovered (immune) (R), characterizing the states of their health. While standard SIR-type models are able to capture the main features of a certain class of infectious diseases such as mumps, measles, and rubella, they fail to describe persistent oscillatory behaviors and spontaneous outbursts, which are observed in many epidemics.

A large amount of work still is devoted to compartmental models [3,4], where an impressive field has emerged [5,6], and the interest was again considerably enhanced by the context of the COVID-19 pandemic [7]. In addition to purely macroscopic models, the study of epidemic dynamics in complex networks has attracted considerable attention [8,9,10]. In these works, the importance of the graph topology for spreading phenomena has been highlighted. In particular, Pastor-Satorras and Vespignani showed that, for a wide range of scale-free networks, no critical threshold for epidemic spreading exists [9]. The topological features crucial for epidemic spreading include the small world property (short average network distances (the network “distance” of two nodes is the number of edges of the shortest path connecting them) and a high clustering coefficient, measuring the existence of redundant paths between pairs of nodes [11,12].

Further interesting directions are represented by combinations of network science and stochastic compartmental models [13,14,15,16,17]. Such models include Markovian and non-Markovian approaches [18,19,20,21,22], where non-Markovianity is introduced by non-exponentially distributed sojourn times in the compartments [23,24]. In these works, explicit formulae for the endemic equilibrium in terms of mean compartmental sojourn times and the basic reproduction number are derived and numerically validated in random walk simulations. A further non-Markovian model appeared recently [25], where non-Markovianity comes into play by introducing an “age of infection”, allowing individuals to recover when their infection period exceeds a certain threshold, generalizing the initial idea of Kermack and McKendrick.

Other works emphasize the importance of the spatial heterogeneity effects of infection patterns in epidemic spreading phenomena [26]. The role of local clusters in generating periodic epidemic outbursts has been highlighted in the references [27,28]. A cluster model to explain periodic behavior was introduced a long time ago [29]. The role of the complex interplay of retardation (delayed compartmental transitions) and fluctuations for oscillatory behavior has been investigated in one of our recent works [22].

The aim of the present paper is to study the spreading of a certain class of vector-transmitted diseases in a population of individuals (random walkers) moving on complex graphs aiming to mimic human mobility patterns in complex environments such as cities, streets, and transportation networks. Essential elements in our model are the accounting of the mortality of infected individuals (random walkers) and an indirect transmission pathway via vectors (nodes of the network). The random walkers are mimicking individuals that are navigating on the network. The nodes of the network are assumed to mimic the vectors (for instance, the mosquitoes in the case of Malaria). The reason why we chose the nodes of the network to mimic the vectors is based on the observation that, in real-world situations, the vectors live in stationary well-defined areas such as swamps and others. The nodes are immortal, since we assume that the vectors (mosquitoes) are not falling ill during their infection time span.

The present paper is organized as follows. In Section 2, we establish a stochastic mean-field model for the evolution of the compartmental populations. The special case of zero mortality is considered in Section 3, where we obtain an explicit formula for the endemic equilibrium (stationary constant compartmental populations for infinite time). In this way, we identify a crucial parameter controlling the stability of the healthy state having the interpretation of the basic reproduction number $R_{0}$ (Section 4), where the healthy state is stable for $R_{0}<1$ and unstable for $R_{0}>1$. A detailed proof of the stability of the endemic state for $R_{0}>1$ is provided in Appendix A.2. In Section 5, we analyze the stability of the healthy state with mortality, derive the basic reproduction number $R_{M}$, and prove that $R_{M}<R_{0}$, i.e., mortality reduces the basic reproduction number. In Section 6, we test the robustness of our mean-field model under “complex real-world conditions” by implementing its assumptions in multiple random random walker simulations on Barabási–Albert (BA)-, Erdös–Rényi (ER)-, and Watts–Strogatz (WS)-type graphs (see [14,15,30] and Appendix A.4). These graph types have different complexity and connectivity features with an impact on the spreading.

## 2. Compartmental Model with Mortality

The goal of this section is to develop a mean-field model for a certain class of diseases with indirect infection transmission via vectors, which includes Dengue, Malaria (transmission by mosquitoes), pestilence (transmission by fleas), and others [8,31]. To that end, we consider a population of *Z* random walkers navigating independently on a connected (ergodic) graph. Each walker performs independent steps from one to another connected node on the network (specified subsequently). We assume that walkers and nodes are in one of the compartments, S (susceptible) and I (infected). In addition, walkers can be in compartment D (dead), whereas nodes never die.

Let $Z_{S}\left(t\right),Z_{I}\left(t\right)$ ($N_{S}\left(t\right),N_{I}\left(t\right)$) be the number of walkers (nodes) in compartments S and I and $Z_{D}\left(t\right)$ the non-decreasing number of walkers (in compartment D) that died from the disease up to time *t*. We consider $Z=Z_{I}\left(t\right)+Z_{S}\left(t\right)+Z_{D}\left(t\right)$ walkers (*Z* independent of time) and a constant number of nodes $N=N_{I}\left(t\right)+N_{S}\left(t\right)$. We assume at instant t=0 the first spontaneous occurrence of the disease of a few infected walkers $Z_{I}\left(0\right)\ll Z$ or nodes $N_{I}\left(0\right)\ll N$ (and no dead walkers $Z_{D}\left(0\right)=0$). We introduce the compartmental fractions $S_{w}\left(t\right)=\frac{Z_{S}\left(t\right)}{Z}$, $J_{w}\left(t\right)=\frac{Z_{I}\left(t\right)}{Z}$, $d_{w}\left(t\right)=\frac{Z_{D}\left(t\right)}{Z}$ for the walkers (normalized with respect to *Z*) with $S_{w}\left(t\right)+J_{w}\left(t\right)+d_{w}\left(t\right)=1$ and $S_{n}\left(t\right)=\frac{N_{S}\left(t\right)}{N}$, $J_{n}\left(t\right)=\frac{N_{I}\left(t\right)}{N}$ with $S_{n}\left(t\right)+J_{n}\left(t\right)=1$. To limit the complexity of our model, we do not consider the demographic evolution, i.e., there are no natural birth and death processes. We denote with $A_{w}\left(t\right),A_{n}\left(t\right)$ the infection rates (rates of transitions S → I) of walkers and nodes, respectively. We assume the following simple bi-linear forms:

(1) $$\begin{array}{ccc} A_{w}\left(t\right) & =A_{w}\left[S_{w}\left(t\right),J_{n}\left(t\right)\right]=\beta_{w}S_{w}\left(t\right)J_{n}\left(t\right) & \\ A_{n}\left(t\right) & =A_{n}\left[S_{n}\left(t\right),J_{w}\left(t\right)\right]=\beta_{n}S_{n}\left(t\right)J_{w}\left(t\right) \end{array}$$

with constant rate parameters $\beta_{w},\beta_{n}>0$ (independent of time). $A_{w}\left(t\right)$ indicates the infection rate of walkers, where its dependence on $S_{w},J_{n}$ is telling us that susceptible walkers can be infected only by (visiting) infected nodes. $A_{n}\left(t\right)$ stands for the infection rate of nodes depending on $S_{n}\left(t\right),J_{w}\left(t\right)$ indicating that susceptible nodes can only be infected by (visits of) infected walkers. There are no direct transmissions among walkers and among nodes. Infections of walkers (nodes) take place with specific transmission probabilities in the contact of a node and a walker, which are captured by (yet not identical to) the transmission rate constants $\beta_{w,n}$.

The infection time spans $t_{I}^{w,n}>0$ without mortality (waiting times in compartment I) of walkers and nodes are assumed to be independent random variables drawn from specific distributions specified hereafter. As the only admitted death process, we assume that infected walkers may die within the time span of their infection. To capture this kind of mortality caused by the disease, we introduce a further independent random variable $t_{M}>0$, which indicates the life span of an infected walker. Both the infection and life spans $t_{I}^{w},t_{M}$ are counted from the time instant of the infection. A walker survives the disease if $t_{M}>t_{I}^{w}$ and dies from it for $t_{M}<t_{I}^{w}$. With these assumptions, we first give a stochastic formulation of the evolution equations:

(2) $$\begin{array}{ccc} \frac{d}{dt}S_{w}\left(t\right) & =-A_{w}\left(t\right)+\langle A_{w}\left(t-t_{I}^{w}\right)\Theta \left(t_{M}-t_{I}^{w}\right)\rangle +J_{w}\left(0\right)\langle \delta \left(t-t_{I}^{w}\right)\Theta \left(t_{M}-t_{I}^{w}\right)\rangle & \\ \frac{d}{dt}J_{w}\left(t\right) & =A_{w}\left(t\right)-\langle \;A_{w}\left(t-t_{I}^{w}\right)\Theta \left(t_{M}-t_{I}^{w}\right)\;\rangle -J_{w}\left(0\right)\langle \;\delta \left(t-t_{I}^{w}\right)\Theta \left(t_{M}-t_{I}^{w}\right)\;\rangle -\frac{d}{dt}d_{w}\left(t\right) & \\ \frac{d}{dt}d_{w}\left(t\right)= & \langle \;A_{w}\left(t-t_{M}\right)\Theta \left(t_{I}^{w}-t_{M}\right)\;\rangle +J_{w}\left(0\right)\langle \delta \left(t-t_{M}\right)\Theta \left(t_{I}^{w}-t_{M}\right)\rangle \; & \\ \frac{d}{dt}S_{n}\left(t\right) & =-A_{n}\left(t\right)+\langle A_{n}\left(t-t_{I}^{n}\right)\rangle +J_{n}\left(0\right)\langle \delta \left(t-t_{I}^{n}\right)\rangle & \\ \frac{d}{dt}J_{n}\left(t\right) & =-\frac{d}{dt}S_{n}\left(t\right) \end{array}$$

where $\frac{d}{dt}d_{w}\left(t\right)$ indicates the (non-negative) mortality rate of walkers. We indicate with $\langle ..\rangle$ the average over the contained (set of independent) random variables $t_{I}^{w},t_{I}^{n},t_{M}$ outlined hereafter and in Appendix A.1. Θ(..) stands for the Heaviside function (A2), and δ(..) for Dirac’s δ-distribution. An epidemic always starts from “natural” initial conditions $S_{w}\left(0\right)=1$, $S_{n}\left(0\right)=1$ (globally healthy state), where, at t=0, the first infections occur spontaneously and can be “generated” by adding the source terms $J_{w,n}\left(0\right)\delta \left(t\right)$ to the infection rates of walkers and nodes, respectively. Equivalently, we introduce initial conditions $S_{w,n}\left(0\right)=1-J_{w,n}\left(0\right)$ ($d_{w}\left(0\right)=0$) with $J_{w,n}\left(0\right)>0$ consisting typically of a few infected walkers and/or nodes in a large susceptible population without dead walkers $d_{w}\left(0\right)=0$.

The interpretation of the system (2) is as follows. The instantaneous infection rate $A_{w}\left(t\right)$ governs the transitions S → I of walkers (due to the visits of infected nodes). The term $\langle \;A_{w}\left(t-t_{I}^{w}\right)\Theta \left(t_{M}-t_{I}^{w}\right)\;\rangle$ describes the rate of walkers recovering at time *t* and infected at $t-t_{I}^{w}$, i.e., their infection time span has elapsed and they survived as $t_{M}>t_{I}^{w}$ (indicated by $\Theta (t_{M}-t_{I}^{w})=1$). Then, $\langle \;A_{w}\left(t-t_{M}\right)\Theta \left(t_{I}^{w}-t_{M}\right)\;\rangle$ captures the rate of walkers infected at $t-t_{M}$ dying at time *t* during the infection time span (indicated by $\Theta (t_{I}^{w}-t_{M})=1$ for $t_{I}^{w}>t_{M}$).

**Remark** **1.** *The infection time span of a walker (sojourn time in compartment I) is $min(t_{I}^{w},t_{M})$, i.e., $t_{I}^{w}$ if $t_{M}>t_{I}^{w}$ (where the walker survives the disease), and is $t_{M}$ if the walker dies within the infectious time span ($t_{M}<t_{I}^{w}$). $t_{I}^{w}$ is the walker’s infection time span without mortality (retrieved for $t_{M}\to \infty$). The probability of the persistence of a walker’s infection at time t, given the infection starts at time 0, is $\langle \;\Theta \left(t_{I}^{w}-t\right)\Theta \left(t_{M}-t\right)\;\rangle$ (see (7)). Note that $\Theta \left(t_{I}^{w}-t\right)\Theta \left(t_{M}-t\right)=1$ only if $t<min(t_{I}^{w},t_{M})$, i.e., when the walker is in compartment I. As a crucial element of our model, we will analyze the statistics of the walker’s infection time span $min(t_{I}^{w},t_{M})$.*

The initially infected walkers and nodes are as well subjected to the transition pathways, i.e., walkers either recover (alive) with rate $J_{w}\left(0\right)\langle \delta \left(t-t_{I}^{w}\right)\Theta \left(t_{M}-t_{I}^{w}\right)\rangle$ or they die with rate $J_{w}\left(0\right)\langle \delta \left(t-t_{M}\right)\Theta \left(t_{I}^{w}-t_{M}\right)\rangle$, and nodes always recover with rate $J_{n}\left(0\right)\langle \delta \left(t-t_{I}^{n}\right)\rangle$. For t→∞, these terms are evanescent; thus, the initial conditions do not affect large time limits (endemic state for zero mortality). The importance of these terms can be seen by setting $\beta_{w,n}=0$ (no infections). Without these terms, the initially infected walkers and nodes would stay infected forever, inconsistent with our assumptions.

The rate equations for the nodes can be interpreted in the same way as the interplay of instantaneous infections and delayed recovery without mortality. We emphasize that the evolution equations of the nodes and walkers are non-linearly coupled by the implicit dependencies of the infection rates (1). In order to derive an explicit representation of the system (2), we need to take a closer look at the averaging procedures and the involved distributions related to the independent random variables $T=\{t_{I}^{w},t_{I}^{n},t_{M}\}>0$ drawn from specific probability density functions (PDFs), which we define by

(3) Prob[T∈[τ,τ+dτ]=K(τ)dτ,

with their respective PDFs (kernels) $K\left(\tau \right)=\{K_{I}^{w,n}\left(\tau \right),K_{M}\left(\tau \right)\}$, which are normalized $Prob\left[T>0\right]=\int_{0}^{\infty }K\left(\tau \right)d\tau =1$. Then, recall the averaging rule for the (suitable) functions f(T) of the random variable *T*, which we use throughout the paper:

(4) $$\langle f(T)\rangle =\int_{0}^{\infty }K\left(\tau \right)f\left(\tau \right)d\tau ;$$

see also Appendix A.1. An important case is 〈δ(t−T)〉=K(t). Then, by applying (4), we introduce the persistence probabilities of the walker’s (node’s) infection (without mortality):

(5) $$\Phi_{I}^{w,n}\left(t\right)=Prob\left(t_{I}^{w,n}>t\right)=\langle \Theta \left(t_{I}^{w,n}-t\right)\rangle =\int_{t}^{\infty }K_{I}^{w,n}\left(\tau \right)d\tau$$

and the probability of the walker’s survival up to time *t* (given $t_{I}^{w}=\infty$):

(6) $$\Phi_{M}\left(t\right)=Prob\left(t_{M}>t\right)=\langle \Theta \left(t_{M}-t\right)\rangle =\int_{t}^{\infty }K_{M}\left(\tau \right)d\tau .$$

The persistence probabilities fulfill the initial condition $\Phi_{M}\left(0\right)=\Phi_{I}^{w,n}\left(0\right)=1$ corresponding to the normalization of the waiting time PDFs $K\left(\tau \right)=\{K_{I}^{w,n}\left(\tau \right),K_{M}\left(\tau \right)\}$ and are vanishing at infinity $\Phi_{M}\left(\infty \right)=\Phi_{I}^{w,n}\left(\infty \right)=0$. To evaluate the averages in (2), we will use the following quantities:

(7) $$\begin{array}{ccc} \langle \delta (t-T)\rangle & =K\left(t\right),\;T=\left\{t_{I}^{w},t_{I}^{n},t_{M}\right\} & \\ \langle \Theta \left(t_{M}-t\right)\Theta \left(t_{I}^{w}-t\right)\rangle & =\langle \Theta \left(t_{M}-t\right)\rangle \langle \Theta \left(t_{I}^{w}-t\right)\rangle =\Phi_{I}^{w}\left(t\right)\Phi_{M}\left(t\right) & \\ b_{d}\left(t\right)=\langle \delta \left(t-t_{M}\right)\Theta \left(t_{I}^{w}-t_{M}\right)\rangle & =\langle \delta \left(t-t_{M}\right)\rangle \langle \Theta \left(t_{I}^{w}-t\right)\rangle =K_{M}\left(t\right)\Phi_{I}^{w}\left(t\right)\; & \\ b_{r}\left(t\right)=\langle \delta \left(t-t_{I}^{w}\right)\Theta \left(t_{M}-t_{I}^{w}\right)\rangle & =\langle \delta \left(t-t_{I}^{w}\right)\rangle \langle \Theta \left(t_{M}-t\right)\rangle =K_{I}^{w}\left(t\right)\Phi_{M}\left(t\right)\; & \\ b_{d}\left(t\right)+b_{r}\left(t\right)=K_{I,M}^{w}\left(t\right) & =-\frac{d}{dt}\left[\langle \Theta \left(t_{M}-t\right)\rangle \langle \Theta \left(t_{I}^{w}-t\right)\rangle \right]=-\frac{d}{dt}\left[\Phi_{I}^{w}\left(t\right)\Phi_{M}\left(t\right)\right] & \\ \int_{0}^{\infty }K_{I,M}^{w}\left(t\right)dt & =1 & \\ R\left(t\right)=\langle \Theta \left(t-t_{I}^{w}\right)\Theta \left(t_{M}-t_{I}^{w}\right)\rangle & =\int_{0}^{t}b_{r}\left(\tau \right)d\tau =\int_{0}^{t}K_{I}^{w}\left(\tau \right)\Phi_{M}\left(\tau \right)d\tau & \\ D\left(t\right)=\langle \Theta \left(t-t_{M}\right)\Theta \left(t_{I}^{w}-t_{M}\right)\rangle & =\int_{0}^{t}b_{d}\left(\tau \right)d\tau =\int_{0}^{t}K_{M}\left(\tau \right)\Phi_{I}^{w}\left(\tau \right)d\tau & \\ R\left(t\right)+D\left(t\right)=\int_{0}^{t}K_{I,M}^{w}\left(\tau \right)d\tau & =\int_{0}^{t}\left[b_{d}\left(\tau \right)+b_{r}\left(\tau \right)\right]d\tau =1-\Phi_{I}^{w}\left(t\right)\Phi_{M}\left(t\right) & \\ D(\infty )+R(\infty )=1 & \\ \langle A\left(t-t_{I}\right)\Theta \left(t_{M}-t_{I}\right)\rangle & =\langle A\left(t-t_{I}\right)\Phi_{M}\left(t_{I}\right)\rangle =\int_{0}^{t}A\left(t-\tau \right)\Phi_{M}\left(\tau \right)K_{I}\left(\tau \right)d\tau & \\ \langle A\left(t-t_{M}\right)\Theta \left(t_{I}^{w}-t_{M}\right)\rangle & =\langle A\left(t-t_{M}\right)\Phi_{I}^{w}\left(t_{M}\right)\rangle =\int_{0}^{t}A\left(t-\tau \right)\Phi_{I}^{w}\left(\tau \right)K_{M}\left(\tau \right)d\tau \end{array}$$

In these averages, we make use of the independence of the waiting times $t_{M},t_{I}^{w,n}$, and of the causality of A(τ) and the kernels K(τ) (i.e., A(τ),K(τ)=0 for τ<0). Of utmost importance are the “defective” PDFs (DPDFs) $b_{d,r}\left(t\right)$ of death and recovery. “Defective” means that $b_{d,r}\left(t\right)$ are not proper PDFs since they are not normalized to one, but rather to D(∞),R(∞)<1, respectively. Consult [32] for a recent account of defective distributions and related stochastic processes. They have the following interpretation. $b_{d}\left(t\right)dt=K_{M}\left(t\right)\Phi_{I}^{w}\left(t\right)dt$ is the probability of transition I → D within [t,t+dt] of an infected walker (infected at $t^{\prime }=0$). $b_{r}\left(t\right)dt=K_{I}^{w}\left(t\right)\Phi_{M}\left(t\right)dt$ is the probability of transition I → S within [t,t+dt] of a walker infected at $t^{\prime }=0$. Therefore,

(8) $$K_{I,M}^{w}\left(t\right)=b_{r}\left(t\right)+b_{d}\left(t\right)=-\frac{d}{dt}\langle \Theta \left(t_{M}-t\right)\Theta \left(t_{I}^{w}-t\right)\rangle =-\frac{d}{dt}\left[\Phi_{I}^{w}\left(t\right)\Phi_{M}\left(t\right)\right]$$

is non-negative (as are $K_{I}^{w}=-\frac{d}{dt}\Phi_{I}^{w}\geq 0$, $K_{M}=-\frac{d}{dt}\Phi_{M}\geq 0$) and is a proper well-normalized PDF of all exits of walkers from compartment I (i.e., I → S + I → D). Without mortality ($\Phi_{M}\left(t\right)=1$), this PDF retrieves $K_{I,M}^{w}\left(t\right)=K_{I}^{w}\left(t\right)$.

The quantities R(t),D(t) introduced in (7) have the following interpretation. R(t) is the probability that a walker infected at instant 0 is at time *t* in compartment S (i.e., recovered prior or up to time *t*). D(t) is the probability that a walker infected at instant 0 is at time *t* in compartment D (i.e., died prior and up to time *t*). The infinite time limits are important: R(∞) has the interpretation of the overall probability that an infected walker survives the infection, and D(∞) is the overall probability for an infected walker to die from the disease. We refer to D(∞) also as “overall mortality”. It must not be confused with the infinite time limit of the dead walker’s fraction $d_{w}\left(\infty \right)$, which is different from D(∞), as we will see in detail subsequently. A small value D(∞) may cause a high value of $d_{w}\left(\infty \right)$, for instance, for short infectious periods where walkers may be repeatedly infected.

In most cases, not all infected walkers die from their disease (in an infinite observation time); hence, D(∞)<1 (as $b_{d}$ is defective). D(∞)→1 represents the limit of a fatal disease and D(∞)→0 a disease without mortality. R(∞)<1 (as $b_{r}$ is defective) is the complementary probability with D(∞)+R(∞)=1.

With these remarks, the system (2) reads

(9) $$\begin{array}{ccc} \frac{d}{dt}S_{w}\left(t\right) & =-A_{w}\left(t\right)+\int_{0}^{t}A_{w}\left(t-\tau \right)K_{I}^{w}\left(\tau \right)\Phi_{M}\left(\tau \right)d\tau +J_{w}\left(0\right)K_{I}^{w}\left(t\right)\Phi_{M}\left(t\right) & \\ \frac{d}{dt}J_{w}\left(t\right) & =\frac{d}{dt}\int_{0}^{t}A_{w}\left(\tau \right)\Phi_{M}\left(t-\tau \right)\Phi_{I}^{w}\left(t-\tau \right)d\tau -J_{w}\left(0\right)\left[K_{I}^{w}\left(t\right)\Phi_{M}\left(t\right)+K_{M}\left(t\right)\Phi_{I}^{w}\left(t\right)\right] & \\ \frac{d}{dt}S_{n}\left(t\right) & =-A_{n}\left(t\right)+\int_{0}^{t}A_{n}\left(t-\tau \right)K_{I}^{n}\left(\tau \right)d\tau +J_{n}\left(0\right)K_{I}^{n}\left(t\right) & \\ \frac{d}{dt}J_{n}\left(t\right) & =-\frac{d}{dt}S_{n}\left(t\right). & \\ & \; \end{array}$$

The PDF (8) for which a walker leaves compartment I (either by recovery or by death) allows rewriting the second equation of (9) as

(10) $$\frac{d}{dt}J_{w}\left(t\right)=A_{w}\left(t\right)-\int_{0}^{t}A_{w}\left(t-\tau \right)K_{I,M}^{w}\left(\tau \right)d\tau -J_{w}\left(0\right)K_{I,M}^{w}\left(t\right).$$

Worthy of closer consideration is the mortality rate of the infected walkers (representing the total mortality; entry rate into the D compartment):

(11) $$\begin{array}{ccc} \frac{d}{dt}d_{w}\left(t\right)=-\frac{d}{dt}\left(S_{w}\left(t\right)+J_{w}\left(t\right)\right) & =\langle \;A_{w}\left(t-t_{M}\right)\Theta \left(t_{I}^{w}-t_{M}\right)\;\rangle +J_{w}\left(0\right)\langle \delta \left(t-t_{M}\right)\Theta \left(t_{I}^{w}-t_{M}\right)\rangle \; & \\ \; & =\int_{0}^{t}A_{w}\left(t-\tau \right)K_{M}\left(\tau \right)\Phi_{I}^{w}\left(\tau \right)d\tau +J_{w}\left(0\right)K_{M}\left(t\right)\Phi_{I}^{w}\left(t\right) \end{array}$$

where, clearly, $\frac{d}{dt}d_{w}\left(t\right)\geq 0$. Integrating this relation yields the fraction $d_{w}\left(t\right)$ of dead walkers:

(12) $$\begin{array}{ccc} d_{w}\left(t\right) & =1-S_{w}\left(t\right)-J_{w}\left(t\right) & \\ \; & =\int_{0}^{t}A_{w}\left(t-\tau \right)\langle \Theta \left(\tau -t_{M}\right)\Theta \left(t_{I}^{w}-t_{M}\right)\rangle d\tau +J_{w}\left(0\right)\langle \Theta \left(t-t_{M}\right)\Theta \left(t_{I}^{w}-t_{M}\right)\rangle & \\ \; & =\int_{0}^{t}A_{w}\left(t-\tau \right)D\left(\tau \right)d\tau +J_{w}\left(0\right)D\left(t\right). \end{array}$$

An interesting quantity is the cumulative recovery rate of walkers (integrated entry rates of walkers into the S compartment; see the first equation in (2)):

(13) $$\begin{array}{ccc} r_{w}\left(t\right) & =\int_{0}^{t}A_{w}\left(t-\tau \right)\langle \Theta \left(\tau -t_{I}^{w}\right)\Theta \left(t_{M}-t_{I}^{w}\right)\rangle d\tau +J_{w}\left(0\right)\langle \Theta \left(t-t_{I}^{w}\right)\Theta \left(t_{M}-t_{I}^{w}\right)\rangle & \\ & =\int_{0}^{t}A_{w}\left(t-\tau \right)R\left(\tau \right)d\tau +J_{w}\left(0\right)R\left(t\right). \end{array}$$

The quantity $r_{w}\left(t\right)$ records all recovery events of walkers up to time *t*, where individual walkers may suffer repeated infections and recoveries. We observe that (see (7))

(14) $$r_{w}\left(t\right)+d_{w}\left(t\right)=\int_{0}^{t}A_{w}\left(t-\tau \right)\left[1-\Phi_{I}^{w}\left(\tau \right)\Phi_{M}\left(\tau \right)\right]d\tau +J_{w}\left(0\right)\left[1-\Phi_{I}^{w}\left(t\right)\Phi_{M}\left(t\right)\right].$$

Relation (12) records all death events of walkers up to time *t*. Since each walker may die only once, it follows indeed that $d_{w}\left(t\right)\in \left[0,1\right]$. Contrarily, the quantity $r_{w}\left(t\right)$ is not restricted to this interval as walkers may be repeatedly infected and recovered, but due to mortality, eventually only a finite number of times ($r_{w}\left(\infty \right)<\infty$; see (18)). Mortality renders the chain of infection and recovery events transient (due to the defective feature of $b_{r}=K_{I}^{w}\Phi_{M}$). To shed more light on the behavior of $r_{w}\left(t\right)$, consider for a moment zero mortality (R(∞)=1) and t→∞: we then have $A_{w}\left(\infty \right)=\beta_{w}S_{w}^{e}J_{n}^{e}>0$ (shown in Section 3); thus, $r_{w}\left(\infty \right)=\infty$ coming along with an infinite chain of recurrent infection and recovery events (as $b_{r}\left(t\right)$ turns into the proper non-defective PDF $b_{r}=K_{I}^{w}$).

Using (7), we can rewrite (2) in equivalent integral form:

(15) $$\begin{array}{ccc} S_{w}\left(t\right) & =1-J_{w}\left(0\right)\left[\Phi_{M}\left(t\right)\Phi_{I}^{w}\left(t\right)+D\left(t\right)\right]-\int_{0}^{t}A_{w}\left(\tau \right)\left[\Phi_{M}\left(t-\tau \right)\Phi_{I}^{w}\left(t-\tau \right)+D\left(t-\tau \right)\right]d\tau & \\ J_{w}\left(t\right) & =J_{w}\left(0\right)\Phi_{M}\left(t\right)\Phi_{I}^{w}\left(t\right)+\int_{0}^{t}A_{w}\left(\tau \right)\Phi_{M}\left(t-\tau \right)\Phi_{I}^{w}\left(t-\tau \right)d\tau & \\ S_{n}\left(t\right) & =1-J_{n}\left(t\right) & \\ J_{n}\left(t\right) & =J_{n}\left(0\right)\Phi_{I}^{n}\left(t\right)+\int_{0}^{t}A_{n}\left(\tau \right)\Phi_{I}^{n}\left(t-\tau \right)d\tau \end{array}$$

and with (redundant) Equation (12) for the fraction of dead walkers. (15) is a self-consistent system since the infection rates are implicit functions of the unknown susceptible and infected population fractions, i.e., $A_{w}\left(t\right)=A_{w}\left[S_{w}\left(t\right),J_{n}\left(t\right)\right]$, $A_{n}\left(t\right)=A_{w}\left[S_{n}\left(t\right),J_{w}\left(t\right)\right]$ (see (1)). Explore now the infinite time limit of (15), where it is convenient to consider the Laplace-transformed equations. We introduce the Laplace transform (LT) (denoted with a hat) of a function g(t) as

$$\hat{g}\left(\lambda \right)=\int_{0}^{\infty }g\left(t\right)e^{-\lambda t}dt$$

where λ denotes the (suitably chosen) Laplace variable. In order to retrieve infinite time limits, we use the asymptotic feature:

(16) $$g\left(\infty \right)=\lim_{\lambda \to 0}\lambda \;\hat{g}\left(\lambda \right)\;\left(=\lim_{\lambda \to 0}\int_{0}^{\infty }g\left(\frac{\tau }{\lambda }\right)e^{-\tau }d\tau \to g\left(\infty \right)\int_{0}^{\infty }e^{-\tau }d\tau \right).$$

Observing that the LT of $\Phi_{I}^{w}\left(t\right)\Phi_{M}\left(t\right)$ is $\lambda^{-1}\left[1-{\hat{K}}_{I,M}\left(\lambda \right)\right]$ and $\hat{D}\left(\lambda \right)=\lambda^{-1}{\hat{b}}_{d}\left(\lambda \right)$, where ${\hat{b}}_{d}\left(0\right)=D\left(\infty \right)$ (see (7)), we arrive at

(17) $$\begin{array}{ccc} J_{w}\left(\infty \right) & =\lim_{\lambda \to 0}\lambda {\hat{J}}_{w}\left(\lambda \right)=\left[1-{\hat{K}}_{I,M}\left(0\right)\right]\left[J_{w}\left(0\right)+{\hat{A}}_{w}\left(0\right)\right]=0 & \\ J_{n}\left(\infty \right) & =\lim_{\lambda \to 0}\lambda {\hat{J}}_{n}\left(\lambda \right)=\left[1-{\hat{K}}_{n}\left(0\right)\right]\left[J_{n}\left(0\right)+{\hat{A}}_{n}\left(0\right)\right]=0\\ d_{w}\left(\infty \right) & =\lim_{\lambda \to 0}\lambda {\hat{d}}_{w}\left(\lambda \right)=D\left(\infty \right)\left[J_{w}\left(0\right)+{\hat{A}}_{w}\left(0\right)\right], & {\hat{A}}_{w}\left(0\right)=\beta_{w}\int_{0}^{\infty }S_{w}\left(\tau \right)J_{n}\left(\tau \right)d\tau \\ S_{w}\left(\infty \right) & =1-d_{w}\left(\infty \right) & \\ S_{n}\left(\infty \right) & =1 \end{array}$$

where ${\hat{A}}_{w,n}\left(0\right)=\int_{0}^{\infty }A_{w,n}\left(t\right)dt<\infty$. In the same way, one obtains

(18) $$r_{w}\left(\infty \right)=R\left(\infty \right)\left(J_{w}\left(0\right)+{\hat{A}}_{w}\left(0\right)\right).$$

Since D(∞) is non-zero, the asymptotic values $S_{w}\left(\infty \right),d_{w}\left(\infty \right)$ depend on the initial condition $J_{w}\left(0\right)$ and the infection (rate) history. This is no longer true for zero mortality (D(∞)=0) and considered in Section 3. We define the overall probability $P_{D}$ that a walker dies from ($P_{R}$ survives) the disease:

(19) $$P_{D}=\frac{d_{w}\left(\infty \right)}{r_{w}\left(\infty \right)+d_{w}\left(\infty \right)}=D\left(\infty \right),\;P_{R}=1-P_{D}=\frac{r_{w}\left(\infty \right)}{r_{w}\left(\infty \right)+d_{w}\left(\infty \right)}=R\left(\infty \right)$$

consistent with our previous interpretation of D(∞),R(∞), and the ratio:

(20) $$\frac{d_{w}\left(\infty \right)}{r_{w}\left(\infty \right)}=\frac{D(\infty )}{R(\infty )}.$$

The quantities (19) and (20) depend only on the stochastic features of $t_{I}^{w}$ and $t_{M}$. They are independent of the infection rates and initial conditions and, therefore, of the topological properties of the network. In addition, they also do not depend on the stochastic features of the node’s infection time span $t_{I}^{n}$.

### 2.1. Markovian (Memoryless) Case

Generally, the system (9) contains the history of the process (memory), which makes the process non-Markovian. The only exception is when all waiting times are exponentially distributed, namely $\Phi_{I}^{w,n}\left(t\right)=e^{-\xi_{I}^{w,n}t}$, $\Phi_{M}\left(t\right)=e^{-\xi_{M}t}$, where $\langle t_{I}^{w,n}\rangle ={\left(\xi_{I}^{w,n}\right)}^{-1}$ and $\langle t_{M}\rangle ={\left(\xi_{M}\right)}^{-1}$. Then, (9) takes with (15) the memoryless form:

(21) $$\begin{array}{ccc} \frac{d}{dt}S_{w}\left(t\right) & =-\beta_{w}S_{w}\left(t\right)J_{n}\left(t\right)+\xi_{I}^{w}J_{w}\left(t\right) & \\ \frac{d}{dt}J_{w}\left(t\right) & =\beta_{w}S_{w}\left(t\right)J_{n}\left(t\right)-\left(\xi_{I}^{w}+\xi_{M}\right)J_{w}\left(t\right) & \\ \frac{d}{dt}d_{w}\left(t\right) & =\xi_{M}J_{w}\left(t\right) & \\ \frac{d}{dt}S_{n}\left(t\right) & =-\beta_{n}S_{n}\left(t\right)J_{w}\left(t\right)+\xi_{I}^{n}J_{n}\left(t\right) & \\ \frac{d}{dt}J_{n}\left(t\right) & =\beta_{n}S_{n}\left(t\right)J_{w}\left(t\right)-\xi_{I}^{n}J_{n}\left(t\right). \end{array}$$

Putting the left-hand sides to zero yields the stationary state:

(22) $$\begin{array}{ccc} J_{w}\left(\infty \right)=J_{n}\left(\infty \right)=A_{w}\left(\infty \right)=A_{n}\left(\infty \right)=0 & \; & \\ S_{w}\left(\infty \right)=1-d_{w}\left(\infty \right), & d_{w}\left(\infty \right)=\xi_{M}\int_{0}^{\infty }J_{w}\left(\tau \right)d\tau & \\ S_{n}\left(\infty \right)=1. & \; \end{array}$$

Let us check whether this result is consistent with (17). To this end, we integrate the second equation in (21) knowing that $J_{w}\left(\infty \right)=0$, leading to

(23) $$0=J_{w}\left(0\right)+\int_{0}^{\infty }A_{w}\left(t\right)dt-\left(\xi_{I}^{w}+\xi_{M}\right)\int_{0}^{\infty }J_{w}\left(t\right)dt;$$

thus, $\int_{0}^{\infty }J_{w}\left(t\right)dt=\frac{1}{\xi_{M}+\xi_{I}^{w}}\left(J_{w}\left(0\right)+{\hat{A}}_{w}\left(0\right)\right)$. Plugging this relation into (22) and accounting for $D\left(\infty \right)=\frac{\xi_{M}}{\xi_{I}^{w}+\xi_{M}}$, we recover indeed the representation of the expression (17).

For zero mortality $\xi_{M}=0$, one can straightforwardly obtain the constant endemic equilibrium values $J_{w}^{e},J_{n}^{e}$ by setting the left-hand side of (21) to zero, leading to the subsequent Equation (32) derived in Section 3 for general waiting time distributions with finite means.

### 2.2. A Few More Words on Waiting Time Distributions

**Figure 1:**
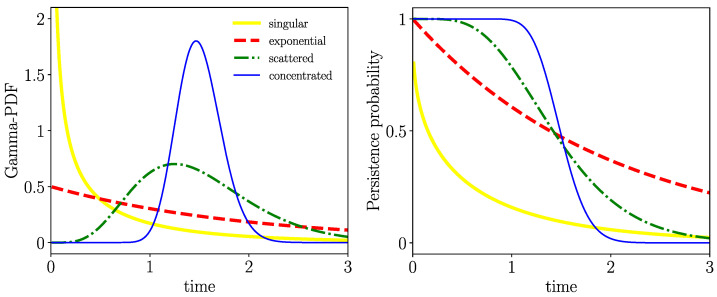
**Left** frame: Gamma distribution for four different cases: weakly singular (at t=0) [〈t〉=0.5, ξ=0.7], exponential [〈t〉=2, ξ=0.5], broad [〈t〉=1.5, ξ=4], and narrow [〈t〉=1.5, ξ=30]. **Right** frame: Their persistence (survival) probability distributions of Equation (27), where the same color code is used.

In our simulations, we assumed that the time spans $t_{I}^{w},t_{I}^{n},t_{M}$ are independent random variables drawn from specific Gamma distributions. The advantage of using Gamma distributions is that they may realize a large variety of shapes; see Figure 1 for a few examples. To generate Gamma-distributed random numbers, we employed the Python 3.10.8 random number generator (library *numpy.random*). Recall the Gamma distribution:

(24) $$K_{\alpha ,\xi }\left(t\right)=\frac{\xi^{\alpha }t^{\alpha -1}}{\Gamma (\alpha )}e^{-\xi t},\;\xi ,\;\alpha >0$$

where α is the so-called “shape parameter” and ξ the rate parameter (often, the term “scale parameter” is used, $\theta =\xi^{-1}$) and Γ(α) stands for the Gamma function. It is worthy of mention that the Gamma distribution for α∈N (also referred to as the Erlang distribution) is the PDF of the sum of independent and identically exponentially distributed random variables. We also will subsequently use the LT of the Gamma PDF:

(25) $${\hat{K}}_{\alpha ,\xi }\left(\lambda \right)=\int_{0}^{\infty }K_{\alpha ,\xi }\left(t\right)e^{-\lambda t}dt=\frac{\xi^{\alpha }}{{\left(\lambda +\xi \right)}^{\alpha }}$$

The Gamma PDF has finite mean ${\langle t\rangle }_{\alpha ,\xi }=\int_{0}^{\infty }tK_{\alpha ,\xi }\left(t\right)dt=\frac{\alpha }{\xi }$, and for α<1, the Gamma PDF is weakly singular at t=0 and α=1 recovers exponential PDFs. For α≤1, the Gamma PDF is completely monotonic (CM) (see Appendix, (A9), for a definition). For the range α>1, the Gamma PDF has a maximum at $t_{max}=\frac{\alpha -1}{\xi }$ and becomes narrower the larger ξ (while keeping its mean α/ξ fixed); specifically, we can generate sharp waiting times using the feature:

(26) $$\lim_{\xi \to \infty }K_{\alpha =\xi T_{0},\xi }\left(t\right)=\delta \left(t-T_{0}\right).$$

We also will subsequently use the persistence probability of the Gamma distribution (see the right frame of Figure 1):

(27) $$\Phi_{\alpha ,\xi }\left(t\right)=\int_{t}^{\infty }\frac{\xi^{\alpha }t^{\alpha -1}}{\Gamma (\alpha )}e^{-\xi t}dt=\frac{\Gamma (\alpha ,\xi t)}{\Gamma (\alpha )}$$

where Γ(α,x) indicates the upper incomplete Gamma function with Γ(α,0)=Γ(α). (27) necessarily fulfills the initial condition $\Phi_{\alpha ,\xi }\left(0\right)=1$ and is vanishing at infinity $\Phi_{\alpha ,\xi }\left(\infty \right)=0$.

## 3. Endemic Equilibrium for Zero Mortality

Here, we consider the large time asymptotics of the compartment populations without mortality ($S_{w}\left(t\right)+J_{w}\left(t\right)=1$), where the self-consistent system (15) reads

(28) $$\begin{array}{ccc} J_{w}\left(t\right) & =J_{w}\left(0\right)\Phi_{I}^{w}\left(t\right)+\int_{0}^{t}A_{w}\left(t-\tau \right)\Phi_{I}^{w}\left(\tau \right)d\tau & \\ J_{n}\left(t\right) & =J_{n}\left(0\right)\Phi_{I}^{n}\left(t\right)+\int_{0}^{t}A_{n}\left(t-\tau \right)\Phi_{I}^{n}\left(\tau \right)d\tau \end{array}$$

The endemic state emerging in the large time asymptotics does not depend on the initial conditions $J_{w,n}\left(0\right)$ as $\Phi_{I}^{w,n}\left(t\right)\to 0$. For what follows, it is convenient to consider the LTs of (28), which read

(29) $$\begin{array}{ccc} {\hat{J}}_{w}\left(\lambda \right) & =\left[J_{w}\left(0\right)+{\hat{A}}_{w}\left(\lambda \right)\right]\frac{1-{\hat{K}}_{I}^{w}\left(\lambda \right)}{\lambda } & \\ {\hat{J}}_{n}\left(\lambda \right) & =\left[J_{n}\left(0\right)+{\hat{A}}_{n}\left(\lambda \right)\right]\frac{1-{\hat{K}}_{I}^{n}\left(\lambda \right)}{\lambda } \end{array}$$

where $\Phi_{I}^{w,n}\left(\lambda \right)=\frac{1-{\hat{K}}_{I}^{w,n}\left(\lambda \right)}{\lambda }$ are the LTs of the persistence distributions and ${\hat{S}}_{w,n}\left(\lambda \right)+{\hat{J}}_{w,n}\left(\lambda \right)=\frac{1}{\lambda }$, reflecting constant populations of walkers and nodes. In order to determine the endemic equilibrium, we assume that the mean infection time spans for the nodes and walkers exist:

(30) $$\langle t_{I}^{w,n}\rangle =\lim_{\lambda \to 0}\frac{1-{\hat{K}}_{I}^{w,n}\left(\lambda \right)}{\lambda }=-\frac{d}{d\lambda }{\hat{K}}_{I}^{w,n}\left(\lambda \right){|}_{\lambda =0}=\int_{0}^{\infty }\Phi_{I}^{w,n}\left(t\right)dt=\int_{0}^{\infty }\tau K_{I}^{w,n}\left(\tau \right)d\tau <\infty ;$$

thus, the admissible range of the Laplace variable is λ≥0 (if chosen real). Now, using (16), we obtain the endemic equilibrium from $J_{w,n}\left(\infty \right)=\lim_{\lambda \to 0}\lambda {\hat{J}}_{w,n}\left(\lambda \right)$, where the initial conditions are wiped out at infinity as ${\hat{K}}_{w,n}\left(\lambda \right){|}_{\lambda =0}=1$. Assuming that the infection rates are constant in the endemic equilibrium, we have $A_{w,n}\left(\lambda \right)∼A_{w,n}\left(\infty \right)/\lambda$, (λ→0) and arrive at

(31) $$\begin{array}{ccc} J_{w}\left(\infty \right) & =A_{w}\left(\infty \right)\langle t_{I}^{w}\rangle ,\;\left(A_{w}\left(\infty \right)=\beta_{w}S_{w}\left(\infty \right)J_{n}\left(\infty \right)\right) & \\ J_{n}\left(\infty \right) & =A_{n}\left(\infty \right)\langle t_{I}^{n}\rangle ,\;\left(A_{n}\left(\infty \right)=\beta_{n}S_{n}\left(\infty \right)J_{w}\left(\infty \right)\right); & \\ & \; \end{array}$$

thus,

(32) $$\begin{array}{cc} \frac{J_{w}\left(\infty \right)}{1-J_{w}\left(\infty \right)}-\beta_{w}\langle t_{I}^{w}\rangle J_{n}\left(\infty \right)=0\; & \\ \frac{J_{n}\left(\infty \right)}{1-J_{n}\left(\infty \right)}-\beta_{n}\langle t_{I}^{n}\rangle J_{w}\left(\infty \right)=0. \end{array}$$

One can see that the globally healthy state $J_{w,n}\left(0\right)=0$ is also a solution of this equation. Besides that, only solutions $J_{n}\left(\infty \right),J_{w}\left(\infty \right)\in \left(0,1\right)$ correspond to an endemic equilibrium. One obtains

(33) $$\begin{array}{c} J_{w}\left(\infty \right)=J_{w}^{e}=\frac{\beta_{w}\beta_{n}\langle t_{I}^{w}\rangle \langle t_{I}^{n}\rangle -1}{\beta_{n}\langle t_{I}^{n}\rangle \left[1+\beta_{w}\langle t_{I}^{w}\rangle \right]}=\frac{R_{0}-1}{R_{0}}\frac{\beta_{w}\langle t_{I}^{w}\rangle }{1+\beta_{w}\langle t_{I}^{w}\rangle }\\ J_{n}\left(\infty \right)=J_{n}^{e}=\frac{\beta_{w}\beta_{n}\langle t_{I}^{w}\rangle \langle t_{I}^{n}\rangle -1}{\beta_{w}\langle t_{I}^{w}\rangle \left[1+\beta_{n}\langle t_{I}^{n}\rangle \right]}=\frac{R_{0}-1}{R_{0}}\frac{\beta_{n}\langle t_{I}^{n}\rangle }{1+\beta_{n}\langle t_{I}^{n}\rangle } \end{array}$$

for the endemic equilibrium, which is independent of the initial conditions $J_{w,n}\left(0\right)$. It depends only on the phenomenological rate constants $\beta_{w,n}$ and the mean infection time spans $\langle t_{I}^{w,n}\rangle$. We point out that the endemic equilibrium (33) has exchange symmetry w↔n between walkers and nodes, reflecting this feature in the system (2) of evolution equations without mortality. The endemic values $J_{w,n}^{e}$ are within (0,1), i.e., existing only if $R_{0}=\beta_{w}\beta_{n}\langle t_{I}^{w}\rangle \langle t_{I}^{n}\rangle >1$. We further mention the useful relation $R_{0}S_{w}\left(\infty \right)S_{n}\left(\infty \right)=1$ following straightforwardly from (32), connecting $R_{0}$ directly with the endemic state. We interpret $R_{0}$ as the basic reproduction number (average number of new infections at t=0—nodes or walkers—due to one infected node or walker at t=0). That this is really the appropriate interpretation can be seen by the following somewhat rough consideration of the infection rates at t=0. Assume we have initially one single infected node $J_{n}\left(0\right)=1/N$ and no infected walkers $S_{w}\left(0\right)=1$. The expected number of walkers infected by this first infected node during its infectious period $t_{I}^{n}$ is

$$\langle Z_{I}\left(t_{I}^{n}\right)\rangle ∼ZA_{w}\left(0\right)\langle t_{I}^{n}\rangle =Z\langle t_{I}^{n}\rangle \beta_{w}/N∼Z\langle J_{w}\left(t_{I}^{w}\right)\rangle .$$

The number of nodes getting infected by these $\langle Z_{I}\left(t_{I}^{n}\right)\rangle$ infected walkers during their infectious time $t_{I}^{w}$ is

$$N_{I}\left(\langle t_{I}^{n}\rangle +\langle t_{I}^{w}\rangle \right)∼N\langle A_{n}\left(t_{I}^{n}\right)\rangle \langle t_{I}^{w}\rangle ∼N\beta_{n}\langle J_{w}\left(t_{I}^{w}\right)\rangle =\beta_{n}\beta_{w}\langle t_{I}^{n}\rangle \langle t_{I}^{w}\rangle =R_{0}.$$

Hence, $R_{0}$ is the average number of infected nodes caused by the first infected node (with zero initially infected walkers). Due to the exchange symmetry (nodes ↔ walkers) of the infection rates, this argumentation remains true when we start with one infected walker and no infected nodes.

We infer that the globally healthy state is unstable for $R_{0}>1$, where the endemic equilibrium (33) is a unique stable fixed point and attractor for all initial conditions $J_{w,n}\left(0\right)$. We will confirm this in the next section by a linear stability analysis of the globally healthy state. The stability of the endemic state is demonstrated in the next section together with Appendix A.2.

The limit $\beta_{w}\langle t_{I}^{w}\rangle \to \infty$ (while $\beta_{n}\langle t_{I}^{n}\rangle$ are kept constant) is remarkable, where all walkers become infected $J_{w}^{e}\to 1$, but not all nodes $J_{n}^{e}\to \frac{\beta_{n}\langle t_{I}^{n}\rangle }{1+\beta_{n}\langle t_{I}^{n}}<1$ and vice versa.

**Figure 2:**
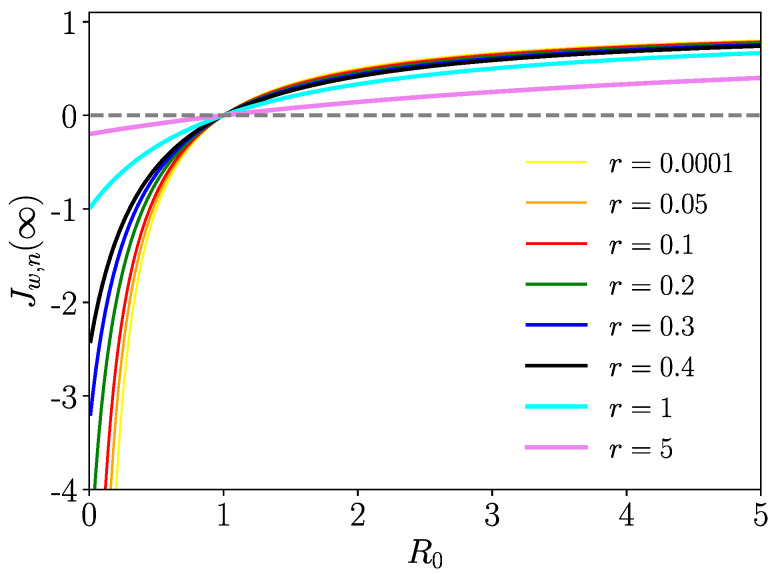
Endemic states of infected walkers/nodes $J_{w,n}\left(\infty \right)=\left(R_{0}-1\right)/\left(R_{0}+r\right)$ versus $R_{0}$ for various values of parameter *r*, which has to be read $r=\beta_{n}\langle t_{I}^{n}\rangle$ ($r=\beta_{w}\langle t_{I}^{w}\rangle$) for the walker’s (node’s) endemic states.

We plot the endemic state in Figure 2 versus $R_{0}$, where positive values for $J_{w,n}\left(\infty \right)$ occur only for $R_{0}>1$, which correspond to endemic states. Next, we perform a linear stability analysis of the endemic and healthy state, where we will indeed identify $R_{0}$ as a crucial control parameter.

## 4. Stability Analysis of Endemic and Healthy State without Mortality

Here, we are interested in the condition of spreading for zero mortality, or equivalently, in the condition for which the globally healthy state (endemic state) is unstable (stable). To check the stability of the endemic fixed point $S_{w}^{e}=1-J_{w}^{e},J_{e}^{w}$, $S_{n}^{e}=1-J_{n}^{e},J_{n}^{e}$, we set

(34) $$\begin{array}{ccc} S_{w}\left(t\right) & =S_{w}^{e}+u_{w}e^{\mu t},\;J_{w}\left(t\right)=J_{w}^{e}-u_{w}e^{\mu t} & \\ S_{n}\left(t\right) & =S_{n}^{e}+u_{n}e^{\mu t},\;J_{n}\left(t\right)=J_{n}^{e}-u_{n}e^{\mu t} \end{array}$$

where $u_{w},u_{n}$ are “small” constant amplitudes. This ansatz accounts for the constant populations of nodes and walkers. Then, we have for the infection rates up to linear orders in the amplitudes:

(35) $$\begin{array}{ccc} A_{w}\left(t\right) & =\beta_{w}S_{w}\left(t\right)J_{n}\left(t\right)=\beta_{w}S_{w}^{e}J_{n}^{e}+\beta_{w}\left(u_{w}J_{n}^{e}-u_{n}S_{w}^{e}\right)e^{\mu t} & \\ A_{n}\left(t\right) & =\beta_{n}S_{n}\left(t\right)J_{w}\left(t\right)=\beta_{n}S_{n}^{e}J_{w}^{e}+\beta_{n}\left(u_{n}J_{w}^{e}-u_{w}S_{n}^{e}\right)e^{\mu t} \end{array}$$

Plugging these relations in our evolution Equation (2) without mortality, omitting two redundant equations leads to the system:

(36) $$\left[\begin{array}{cc} \mu +\beta_{w}J_{n}^{e}\left[1-{\hat{K}}_{I}^{w}\left(\mu \right)\right]; & -\beta_{w}S_{w}^{e}\left[1-{\hat{K}}_{I}^{w}\left(\mu \right)\right]\\ -\beta_{n}S_{n}^{e}\left[1-{\hat{K}}_{I}^{n}\left(\mu \right)\right]; & \mu +\beta_{n}J_{w}^{e}\left[1-{\hat{K}}_{I}^{n}\left(\mu \right)\right] \end{array}\right]\cdot \left(\begin{array}{c} u_{w}\\ u_{n} \end{array}\right)=\left(\begin{array}{c} 0\\ 0 \end{array}\right)$$

where we have used $\langle e^{-\mu t_{I}^{w,n}}\rangle ={\hat{K}}_{I}^{w,n}\left(\mu \right)$ and the cases of δ-distributed $t_{I}^{w,n}$ are contained for ${\hat{K}}_{I}^{w,n}\left(\mu \right)=e^{-\mu t_{I}^{w,n}}$. We point out that, in the ansatz (35), we relax causality, i.e., we admit $A_{w,n}\left(t-\tau \right)\neq 0$ for t−τ<0; thus,

(37) $$\langle e^{\mu (t-t_{I}^{w,n})}\rangle =e^{\mu t}\langle e^{-\mu t_{I}^{w,n}}\rangle =e^{\mu t}{\hat{K}}_{I}^{w,n}\left(\mu \right).$$

The solvability of this matrix equation requires the determinant to vanish, leading to a transcendental characteristic equation for μ:

(38) $$\mu^{2}+\mu \left(\beta_{w}J_{n}^{e}\left[1-{\hat{K}}_{I}^{w}\left(\mu \right)\right]+\beta_{n}J_{w}^{e}\left[1-{\hat{K}}_{I}^{n}\left(\mu \right)\right]\right)+\beta_{w}\beta_{n}\left[1-{\hat{K}}_{I}^{w}\left(\mu \right)\right]\left[1-{\hat{K}}_{I}^{n}\left(\mu \right)\right]\left(J_{n}^{e}J_{w}^{e}-S_{n}^{e}S_{w}^{e}\right)=0.$$

Generally, a fixed point is unstable if solutions with the positive real part of μ exist. Consider this first for the globally healthy state $J_{n}=0,J_{w}=0$ for which Equation (38) reads

(39) $$G\left(\mu \right)=1-\beta_{w}\beta_{n}\frac{\left[1-{\hat{K}}_{I}^{w}\left(\mu \right)\right]}{\mu }\frac{\left[1-{\hat{K}}_{I}^{n}\left(\mu \right)\right]}{\mu }=1-\beta_{w}\beta_{n}{\hat{\Phi }}_{I}^{w}\left(\mu \right){\hat{\Phi }}_{I}^{n}\left(\mu \right)=0$$

where we notice that $\frac{\left[1-{\hat{K}}_{I}^{w,n}\left(\mu \right)\right]}{\mu }={\hat{\Phi }}_{I}^{w,n}\left(\mu \right)$ are the LTs of the persistence probabilities of the infection time spans. Consider this equation for μ→0, and take into account (30); we arrive at

(40) $$G\left(0\right)=1-\beta_{w}\beta_{n}\langle t_{I}^{w}\rangle \langle t_{I}^{n}\rangle .$$

We observe that G(0)<0 for $R_{0}=\beta_{n}\beta_{w}\langle t_{I}^{w}\rangle \langle t_{I}^{n}\rangle >1$. On the other hand, we have for μ→∞ that ${\hat{\Phi }}_{I}^{w,n}\left(\mu \right)\to 0$, and hence,

(41) G(∞)=1.

**Figure 3:**
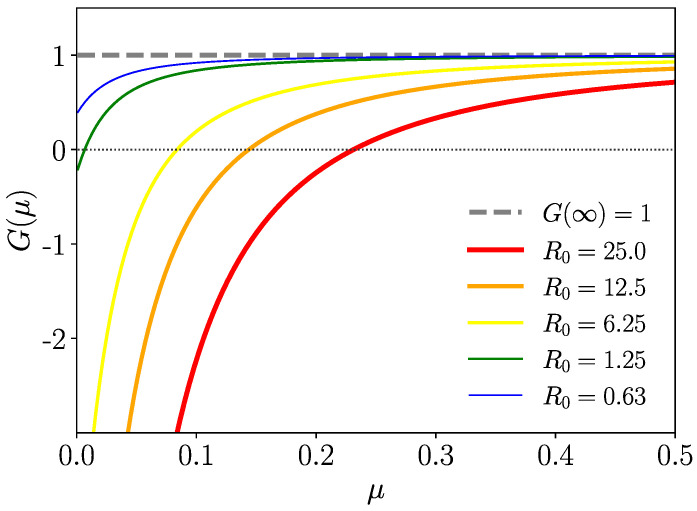
G(μ) of (39) for some Gamma-distributed $t_{I}^{w,n}$. Positive zeros of G(μ) exist only for $R_{0}>1$ (instability of globally healthy state).

One can, hence, infer from the complete monotony of ${\hat{\Phi }}_{I}^{w,n}\left(\mu \right)$ and, therefore, of ${\hat{\Phi }}_{I}^{w}\left(\mu \right){\hat{\Phi }}_{I}^{n}\left(\mu \right)$ (see Appendix A.2, Equation (A9), for a precise definition) that $\frac{d}{d\mu }G\left(\mu \right)>0$ (μ≥0); thus, G(μ)=0 has one single positive zero only if G(0)<0, i.e., for $R_{0}>1$, which, therefore, is the condition of the instability of the healthy state (spreading of the disease). Conversely, for $R_{0}<1$, the healthy state turns into a stable fixed point where there is no spreading of the disease. In particular, the healthy state is always unstable ($R_{0}=\infty$) if at least one of the mean infection time spans $\langle t_{I}^{w,n}\rangle =\infty$. This is true for fat-tailed kernels scaling as $K_{I}^{w,n}\left(t\right)\propto t^{-\alpha -1}$ (α∈(0,1)) for t→∞. We consider such a distribution briefly in the subsequent section. We plot function G(μ) versus μ for different $R_{0}$ for Gamma-distributed $t_{I}^{w,n}$ in Figure 3.

Now, we consider the stability of the endemic state with $G_{e}\left(\mu \right)=0$, where, from (38), this function reads

(42) $$G_{e}\left(\mu \right)=1-\beta_{w}\beta_{n}{\hat{\Phi }}_{I}^{w}\left(\mu \right){\hat{\Phi }}_{I}^{n}\left(\mu \right)+\beta_{w}J_{n}^{e}{\hat{\Phi }}_{I}^{w}\left(\mu \right)+\beta_{n}J_{w}^{e}{\hat{\Phi }}_{I}^{n}\left(\mu \right)+\beta_{w}\beta_{n}\left(J_{w}^{e}+J_{n}^{e}\right){\hat{\Phi }}_{I}^{w}\left(\mu \right){\hat{\Phi }}_{I}^{n}\left(\mu \right)$$

with

(43) $$\begin{array}{ccc} G_{e}\left(0\right) & =1-R_{0}+\beta_{w}\langle t_{I}^{w}\rangle J_{n}^{e}+\beta_{n}\langle t_{I}^{n}\rangle J_{w}^{e}+\left(J_{w}^{e}+J_{n}^{e}\right)R_{0} & \\ \; & =R_{0}-1. \end{array}$$

**Figure 4:**
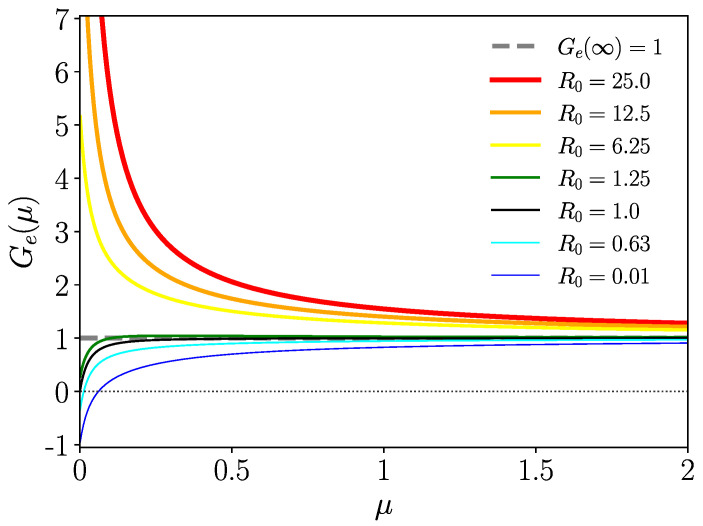
$G_{e}\left(\mu \right)$ of (42) for different values of $R_{0}$, where $G_{e}\left(\mu \right)>0$ for $R_{0}>1$ (stability of the endemic state).

On the other hand, $G_{e}\left(\infty \right)=1$ (as ${\hat{\Phi }}_{I}^{w,n}\left(\infty \right)=0$), and from the monotony of $G_{e}\left(\mu \right)$, it follows that there is no positive solution of $G_{e}\left(\mu \right)=0$ for $R_{0}>1$. We plot $G_{e}\left(\mu \right)$ in Figure 4 for different values of $R_{0}$ and Gamma-distributed $t_{I}^{w,n}$. In Appendix A.2, we complete the analytical proof that $G_{e}\left(\mu \right)>0$ for $R_{0}>1$.

## 5. Stability Analysis of the Healthy State with Mortality

An important question is how mortality modifies the instability of the healthy state and the basic reproduction number. To shed light on this question, we perform a linear stability analysis of the healthy state $S_{w,n}=1$, where we set

(44) $$S_{w}\left(t\right)=1+a\;e^{\mu t},\;J_{w}\left(t\right)=b\;e^{\mu t},\;d_{w}\left(t\right)=-\left(a+b\right)\;e^{\mu t},\;S_{n}\left(t\right)=1-ce^{\mu t},\;J_{n}\left(t\right)=ce^{\mu t}$$

with $A_{w}\left(t\right)=\beta_{w}c\;e^{\mu t}$ and $A_{n}\left(t\right)=\beta_{n}b\;e^{\mu t}$. Plugging this ansatz for μ≥0 into the three independent equations of (2), say the first, third, and fourth one, and performing the averages (relaxing causality as previously), we arrive at

(45) $$\left[\begin{array}{ccc} \mu \;; & \;0; & \beta_{w}\left[1-{\hat{b}}_{r}\left(\mu \right)\right]]\\ \mu ; & \;\mu \;; & \beta_{w}{\hat{b}}_{d}\left(\mu \right)\\ 0\; & -\beta_{n}\left[1-{\bar{K}}_{I}^{n}\left(\mu \right)\right]\;; & \mu \end{array}\right]\cdot \left(\begin{array}{c} a\\ b\\ c \end{array}\right)=\left(\begin{array}{c} 0\\ 0\\ 0 \end{array}\right).$$

Putting the determinant of the matrix to zero yields the condition:

(46) $$\mu^{2}-\beta_{n}\beta_{w}\left[1-{\bar{K}}_{I}^{n}\left(\mu \right)\right]\left[1-{\hat{b}}_{r}\left(\mu \right)-{\hat{b}}_{d}\left(\mu \right)\right]=0$$

where the LTs ${\hat{b}}_{r}\left(\mu \right),{\hat{b}}_{d}\left(\mu \right)$ of the DPDFs $b_{r,d}\left(t\right)$ defined in (7) come into play. We are interested in under which conditions there is a positive solution (instability of the healthy state) of (46). Since ${\hat{b}}_{r}\left(0\right)=R\left(\infty \right)$ and ${\hat{b}}_{d}\left(0\right)=D\left(\infty \right)$ with R(∞)+D(∞)=1, we see that μ=0 is a solution of (46). Recall from (7) that $b_{d}\left(t\right)+b_{r}\left(t\right)=K_{I,M}^{w}\left(t\right)$ is the (properly normalized) PDF (8). Condition (46) then reads

(47) $$G_{M}\left(\mu \right)=1-\beta_{n}\beta_{w}\frac{\left[1-{\bar{K}}_{I}^{n}\left(\mu \right)\right]}{\mu }\frac{\left[1-{\hat{K}}_{I,M}^{w}\left(\mu \right)\right]}{\mu }=0$$

where $\frac{\left[1-{\hat{K}}_{I,M}^{w}\left(\mu \right)\right]}{\mu }$ is the LT of the persistence probability $\Phi_{M}\left(t\right)\Phi_{I}^{w}\left(t\right)$ of the walker’s infection, i.e., the probability that $t<min(t_{I}^{w},t_{M})$ (see Remark 1). For zero mortality, we have $K_{I,M}^{w}=K_{I}^{w}$, ($b_{r}=K_{I}^{w}$ and $b_{d}=0$), retrieving the condition (39). The mean sojourn time in compartment I with mortality yields

(48) $$\langle min\left(t_{I}^{w},t_{M}\right)\rangle =\langle t_{IM}^{w}\rangle =\frac{\left[1-{\hat{K}}_{I,M}^{w}\left(\mu \right)\right]}{\mu }{|}_{\mu =0}=\int_{0}^{\infty }tK_{I,M}^{w}\left(t\right)dt=\int_{0}^{\infty }\Phi_{M}\left(t\right)\Phi_{I}^{w}\left(t\right)dt\;\leq \;\int_{0}^{\infty }\Phi_{I}^{w}\left(t\right)dt=\langle t_{I}^{w}\rangle$$

where we arrive at

(49) $$G_{M}\left(0\right)=1-\beta_{n}\beta_{w}\langle t_{I}^{n}\rangle \langle t_{IM}^{w}\rangle .$$

**Figure 5:**
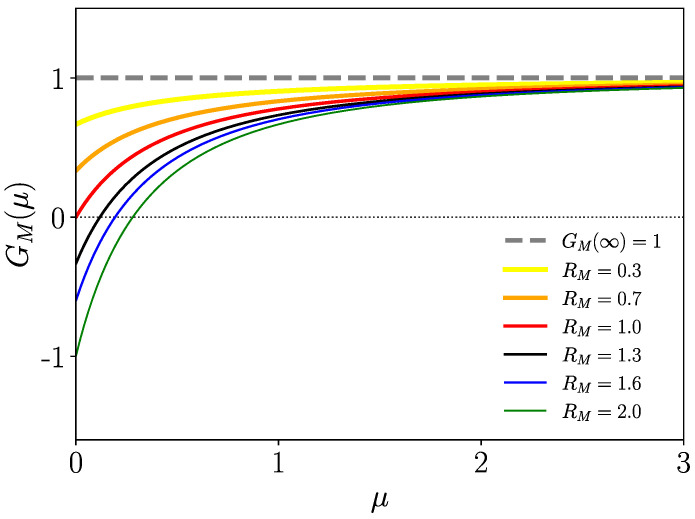
We depict function $G_{M}\left(\mu \right)$ of Equation (47) for a few values of $R_{M}$ for exponentially distributed $t_{I}^{w,n},t_{M}$. The basic reproduction number $R_{M}$ is monotonously decreasing with increasing mortality parameter $\xi_{M}$ (see Figure 6). The parameters are $\beta_{w,n}=1$, $\alpha_{I}^{w}=1$, $\xi_{I}^{w}=1$, $\alpha_{n}=1$, $\xi_{I}^{n}=0.5$ with $R_{0}=2$, where, here, $\langle t_{I,M}\rangle =R_{0}/\left(1+\xi_{M}\right)$.

Relation (48) shows that $\langle t_{IM}^{w}\rangle \leq \langle t_{I}^{w}\rangle$ (equality only for zero mortality). On the other hand, we have $G_{M}\left(\infty \right)=1$, so there is a positive solution of $G_{M}\left(\mu \right)=0$ only if

(50) $$R_{M}=\beta_{n}\beta_{w}\langle t_{I}^{n}\rangle \langle t_{IM}^{w}\rangle \;>\;1$$

where $R_{M}$ is the basic reproduction number modified by mortality with $R_{M}\leq R_{0}$ (equality only for zero mortality). To visualize the effect of mortality on the instability of the healthy state, we plot $G_{M}\left(\mu \right)$ for a few values of $R_{M}$ in Figure 5. Increasing mortality turns an unstable healthy state into a stable one.

**Figure 6:**
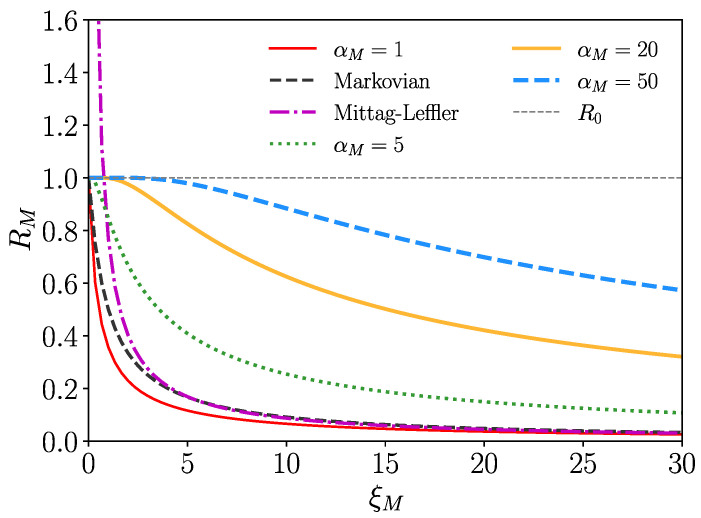
Basic reproduction number $R_{M}$ of Equation (52) versus mortality rate parameter $\xi_{M}$ for Gamma-distributed $t_{I}^{w,n},t_{M}$ for various $\alpha_{M}$, where we have set $\beta_{n}=\beta_{w}=\langle t_{I}^{w}\rangle =\langle t_{I}^{n}\rangle =1$, ($\alpha_{I}^{w}=\xi_{I}^{w}=0.3$) and $\alpha_{M}=1$, $\alpha_{w}=\xi_{I}^{w}=1$ for the Markovian case, which is recovered by Equation (53).

In the random walk simulations, we deal with Gamma-distributed $t_{I}^{w,n},t_{M}$, where the persistence probabilities are then normalized incomplete Gamma functions (27). To explore the effect of mortality for such cases, we determine $R_{M}$ by numerical integration of (48) as a function of the mortality rate parameter $\xi_{M}$ and plot the result in Figure 6, where one can see that $R_{M}$ is monotonously decreasing with mortality rate $\xi_{M}$. We also include a case of a fat-tailed (Mittag–Leffler)-distributed $t_{I}^{w}$, which we discuss hereafter. The parameters in Figure 6 are such that the zero mortality case occurs with $R_{0}=1$ as the upper bound for the Gamma-distributed cases. The essential feature is that $R_{M}$ decays monotonically with increasing mortality rate parameter $\xi_{M}$ approaching zero for $\xi_{M}\to \infty$. Diseases with high mortality stabilize the healthy state even for $\langle t_{I}^{w}\rangle \to \infty$.

Consider briefly the case where $t_{M}$ is exponentially distributed (i.e., $\alpha_{M}=1$ in the Gamma distribution of $t_{M}$) with $\Phi_{M}\left(t\right)=e^{-\xi_{M}t}$. Then, we have $\langle t_{IM}^{w}\rangle ={\hat{\Phi }}_{I}^{w}\left(\xi_{M}\right)$; thus,

(51) $$R_{M}=\beta_{w}\beta_{n}\langle t_{I}^{n}\rangle {\hat{\Phi }}_{I}^{w}\left(\xi_{M}\right).$$

The zero mortality case is recovered for $\xi_{M}=0$ with ${\hat{\Phi }}_{I}^{w}\left(0\right)=\langle t_{I}^{w}\rangle$. For Gamma-distributed $t_{I}^{w,n}$, this yields

(52) $$R_{M}=\beta_{w}\beta_{n}\frac{\alpha_{I}^{n}}{\xi_{I}^{n}\xi_{M}}\left(1-\frac{{\left(\xi_{I}^{w}\right)}^{\alpha_{I}^{w}}}{{\left(\xi_{M}+\xi_{I}^{w}\right)}^{\alpha_{I}^{w}}}\right),$$

where $\alpha_{I}^{w,n}$, $\xi_{I}^{w,n}$ are the parameters of the respective Gamma distributions of the infection times of nodes and walkers. The Markovian case where all waiting times are exponentially distributed is covered for $\alpha_{I}^{w,n}=1$ and yields

(53) $$R_{M}=\frac{\beta_{w}\beta_{n}}{\xi_{I}^{n}\left(\xi_{I}^{w}+\xi_{M}\right)}$$

containing the zero mortality case for $\xi_{M}=0$.

### Fat-Tailed-Distributed $t_{I}^{w}$

Finally, an interesting case emerges if $t_{I}^{w}$ follows a fat-tailed distribution, i.e., $\Phi_{I}^{w}\left(t\right)\propto t^{-\alpha }$ for *t* large (α∈(0,1)) and $\langle t_{I}^{w}\rangle =\infty$, $R_{0}=\infty$. Let us take a look at how mortality is affecting this situation. Fat-tailed $t_{I}^{w}$ distributions describe diseases where the infectious periods are very long and the healthy state without mortality is extremely unstable ($R_{0}=\infty$). Infected walkers can infect many nodes during their long infection time spans. An important case of this class is constituted by the Mittag–Leffler (ML) distribution $\Phi_{I}^{w}\left(t\right)=E_{\alpha }\left(-\xi_{I}^{w}t^{\alpha }\right)$, where $E_{\alpha }\left(\tau \right)$ indicates the Mittag–Leffler function; see [33,34] and the references therein for representations and connections with fractional calculus. The ML function recovers the exponential for α=1 ($E_{1}\left(-\tau \right)=e^{-\tau }$). Assuming exponential mortality $\Phi_{M}\left(t\right)=e^{-\xi_{M}t}$, one obtains with (51)

(54) $$R_{M}=\beta_{w}\beta_{n}\langle t_{I}^{n}\rangle \frac{{\left(\xi_{M}\right)}^{\alpha -1}}{\xi_{I}^{w}+{\left(\xi_{M}\right)}^{\alpha }},\;\alpha \in \left(0,1\right)$$

containing the LT of the ML persistence probability distribution ${\hat{\Phi }}_{I}^{w}\left(\lambda \right)=\lambda^{\alpha -1}/\left(\xi_{I}^{w}+\lambda^{\alpha }\right)$. The essential feature here is that $R_{M}$ is weakly singular at $\xi_{M}=0$ with a monotonously decreasing $\xi_{M}^{\alpha -1}$ scaling law, where the healthy state becomes stable for mortality parameters larger as $\xi_{M}\approx 1$. We depict this case in Figure 6 for α=ξ=0.3 (violet curve).

## 6. Random Walk Simulations

The remainder of our paper is devoted to testing the mean-field model under “real-world conditions”, which we mimic by $Z=Z_{S}\left(t\right)+Z_{I}\left(t\right)+Z_{D}\left(t\right)$ random walkers navigating independently on an undirected connected (ergodic) graph. In our simulations, we focused on Barabási–Albert (BA), Erdös–Rényi (ER), and Watts–Strogatz (WS) graphs [16,17,35] (see Appendix A.4 for a brief recap) and implemented the compartments and transmission pathway for walkers and nodes outlined in Section 2. A susceptible walker gets infected with probability $p_{w}$ by visiting an infected node, and a susceptible node gets infected with probability $p_{n}$ at a visit of an infectious walker. We assumed that the infection probabilities $p_{n,w}$ are constant for all nodes and walkers, respectively. They are related, yet not identical to the macroscopic rate constants $\beta_{w,n}$. A critical issue is whether the simple bi-linear forms for the mean-field infection rates (1) still capture the complexity of the spreading in such “real world” networks well. One goal of the subsequent case study is to explore this question.

We characterize the network topology by i=1,…N nodes with the N×N adjacency matrix $\left(A_{ij}\right)$, where $A_{ij}=1$ if the pair of nodes i,j is connected by an edge and $A_{ij}=0$ if the pair is disconnected. Further, we assumed $A_{ii}=0$ to avoid self-connections of nodes. We confined ourselves to undirected networks, where the edges have no direction and the adjacency matrix is symmetric. The degree $k_{i}$ of a node *i* counts the number of neighbor nodes (edges) of this node. Each walker z=1,…,Z performs simultaneous independent random steps at discrete time instants t=Δt,2Δt,… from one to another connected node. The steps from a node *i* to one of the neighbor nodes are chosen with probability $1/k_{i}$, following for all walkers the same transition matrix:

(55) $$\Pi \left(i\to j\right)=\frac{A_{ij}}{k_{i}},\;z=1,\ldots ,Z,\;i,j=1,\ldots ,N,$$

which is normalized $\sum_{j=1}^{N}\Pi \left(i\to j\right)=1$. This is a common way to connect the network topology with simple Markovian random walks [30,35]. In the simulations, the departure nodes at t=0 of the walkers are randomly chosen. The path of each walker is independent and not affected by contacts with other walkers or by transition events from one to another compartment.

### Case Study and Discussion

In order to compare the epidemic dynamics of the mean-field model and random walk simulations, we integrated the stochastic evolution Equation (2) numerically as follows. We averaged the increments of the compartmental fractions in a generalized Monte Carlo sense converging towards the convolutions of the right-hand side of (9), where we use the Monte Carlo convergence feature:

(56) $$\lim_{n\to \infty }\frac{1}{n}\sum_{k=1}^{n}A\left(t-T_{k}\right)=\int_{0}^{t}A\left(t-\tau \right)K\left(\tau \right)d\tau$$

for random variables *T* drawn from PDFs K(τ). We performed this average for any time increment dt with respect to all involved independent random time spans $t_{I}^{w,n},t_{M}$ (see Appendix A.1) and integrated the averaged compartmental increments in a fourth-order Runge–Kutta scheme (RK4). We used in the random walk simulations and the Monte Carlo (mean-field) integration exactly the same (Gamma-distributed) random values (Python 3.10.8 seeds) for $t_{I}^{w,n},t_{M}$. The values of the infection rate parameters $\beta_{w,n}$ used in the mean-field integration were determined from Equation (32) by plugging in the large time asymptotic values of the random walk simulation with identical parameters (without mortality). The compartmental fractions in the random walk simulations were determined by simply counting the compartmental populations at each time increment Δt of the walker’s steps. The so-determined rate parameters $\beta_{w,n}$ plugged into the mean-field integration depend in a complex manner on the infection probabilities $p_{w,n}$ and topology of the network. In this way, this information is also contained in the basic reproduction numbers with and without mortality. Indeed, the importance of the structural features (topology) of the network is crucial for the spreading of the disease. We refer to the recent review article [36] exploring a large variety of these effects. In that work, a detailed study was performed on the role of structural elements such as the average distance of the nodes and the effective network size (among others) on the epidemic spreading. Naturally, pure mean-field (compartment) approaches ignore the topological features of the environments where the diseases are spreading. In our mean-field model, the structural network properties are captured by the infection rate parameters $\beta_{w,n}$ as outlined above.

**Figure 7:**
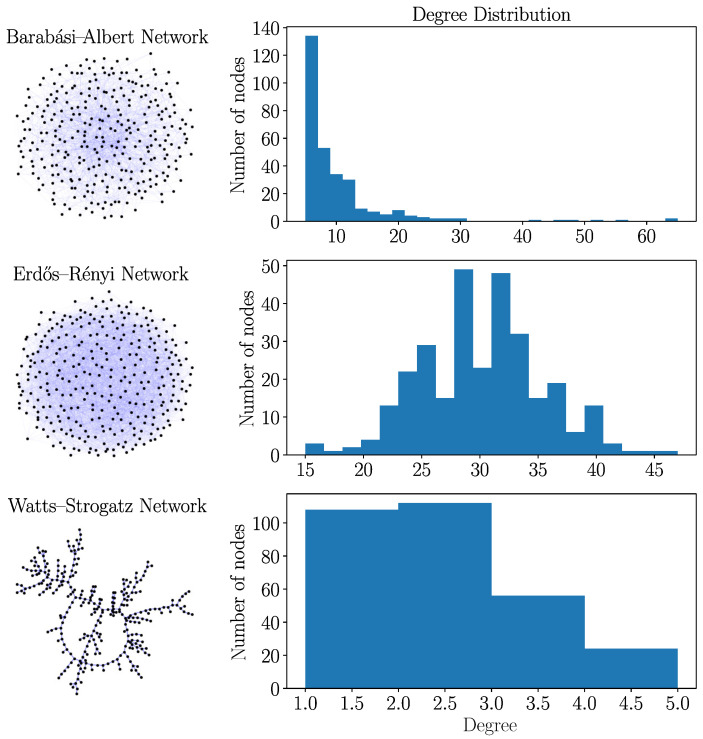
Barabási–Albert, Erdös–Rényi, and Watts–Strogatz types with 300 nodes and connectivity parameters used in some of the simulations. The WS graph for connectivity parameter m=2 lacks the small-world property, resembling a complex real-world structure. The ER network has a broad degree distribution and the small-world property. The BA graph is for N→∞ asymptotically scale-free with a power law degree distribution and the small-world feature, where a large number of nodes have small degrees and, a few (hub) nodes, very large degrees. Almost all nodes are only a few links away from hub nodes.

We explore now the spreading in random graphs of different complexity such as represented in Figure 7. The BA graph is small-world with a power law-distributed degree (Appendix A.4), which means that there are many nodes having a few connections and a few (hub) nodes with a huge number of connections. The average distance between nodes becomes small, as it is sufficient that almost every node is only a few links away from a hub node. The ER graph is small-world due to a broad degree distribution. The WS graph with the choice of connectivity parameter m=2 in Figure 7 has long average distances and is large-world. Intuitively, one infers that a small-world structure is favorable for spreading processes, a feature that was already demonstrated in the literature [8,9]. In our simulations, spreading in network architectures with increased connectivity comes along with increased values of $R_{0}$ and $R_{M}$, respectively.

We identified the starting time instant (t=0) of the evolution in the mean-field model with the time instant of the first infection of a walker in the random walk simulations. In all cases, we started with a small number of randomly chosen initially infected nodes $N_{I}\left(0\right)=10\ll N$ ($N_{I}\left(0\right)\approx 10$) and no infected or dead walkers. To reduce the number of parameters and to concentrate on topological effects, we have put in all simulations the transmission probabilities $p_{w}=p_{n}=1$. We refer to [37] for the Python codes (free to download and non-commercial use) and animated simulation videos related to the present study.

**Figure 8:**
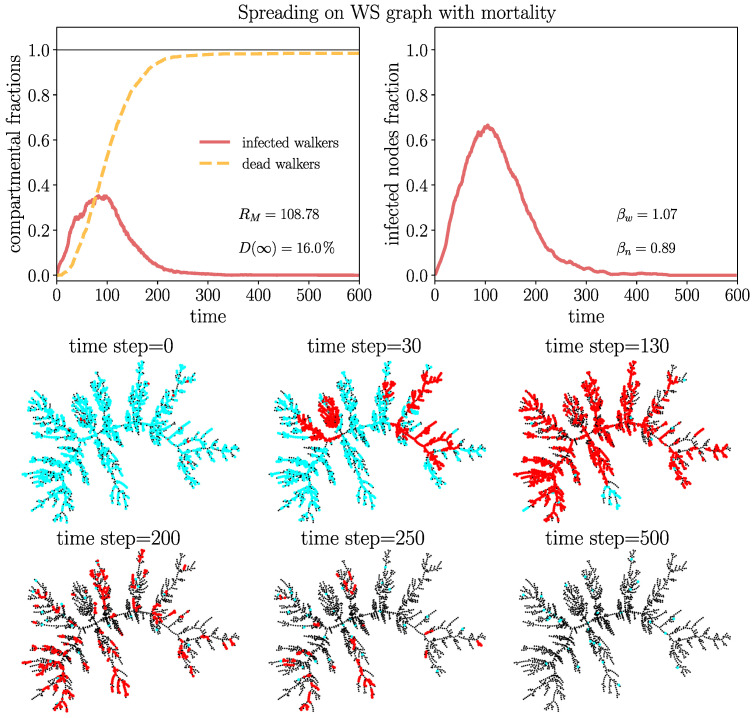
Snapshots of spreading in a WS graph (Z=2000 walkers, N=2000 nodes, connectivity parameter m=2) and mortality parameter $\xi_{M}=0.4$ with D(∞)≈16%. The average degree is $\langle k\rangle =\sum_{i=1}^{N}k_{i}/N=2$, here coinciding with connectivity parameter *m*. Other parameters are the same as in Figure 9. The upper frames show the evolution from the random walk data. One observes $d_{w}\left(\infty \right)\approx 0.99$ and $S_{w}\left(\infty \right)\approx 1\%$ with only about 20 surviving walkers after extinction of the disease. S walkers are drawn in cyan color; I walkers are in red; D walkers are invisible; nodes without walkers are represented in black. Consult here an animated video of this process https://drive.google.com/file/d/1-fhroAsoAVDKGR5H9yWtqjD7A1ZU5pQt/view (accessed on 22 April 2024).

In order to visualize a typical spreading process, we depict in Figure 8 a few snapshots in a Watt–Strogatz graph with a rather high overall mortality probability of D(∞)≈16%. In this case, a single infection wave emerges where a large part of the walkers gets repeatedly infected, increasing their probability to die. This leads to a very high fraction of eventually dead walkers $d_{w}\left(\infty \right)\approx 99\%$ and a small fraction $S_{w}\left(\infty \right)\approx 1\%$ of surviving walkers corresponding to the stationary state (17), which is taken as soon as the disease becomes extinct $J_{w}=J_{n}=0$. Figure 8 shows that, first, the infection gains large parts of the network, consistent with the large value of $R_{M}$ observed in this case. After the first wave, the disease becomes extinct by the high mortality of the walkers. A disease with similar high mortality characteristics is, for instance, pestilence. The process of Figure 8 is visualized in an animated video https://drive.google.com/file/d/1-fhroAsoAVDKGR5H9yWtqjD7A1ZU5pQt/view (accessed on 22 April 2024).

**Figure 9:**
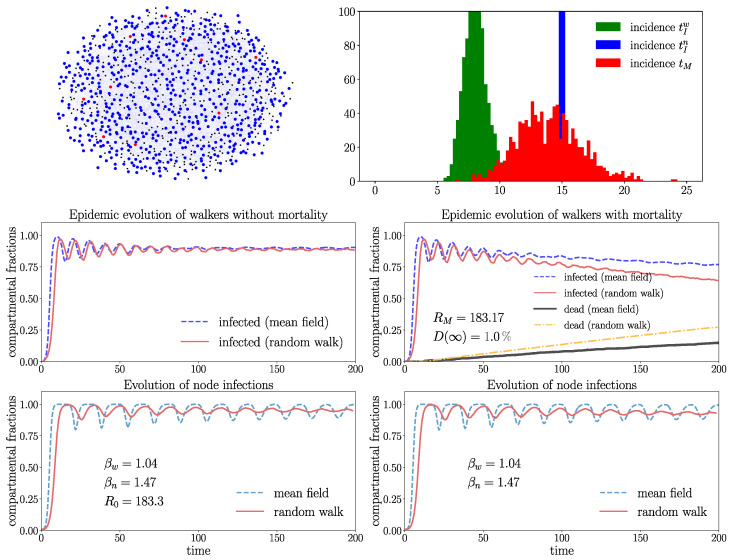
The plots show the evolution on the WS graph with Z=1000 walkers for connectivity parameter m=8 (coinciding with the average degree) and rewiring probability p=0.7 (nx.connected_watts_strogatz_graph(N=1000,m=8,p=0.7)) without mortality (**left** frame) and with mortality (**right** frame). $t_{I}^{w,n},t_{M}$ are Gamma-distributed with the parameters $\langle t_{M}\rangle =14$, $\xi_{M}=2$, $\langle t_{I}^{w}\rangle =8$, $\xi_{I}^{w}=10$, and $\langle t_{I}^{n}\rangle =15$, $\xi_{n}=10^{5}$; see the histogram. The overall mortality D(∞)≈1% is the same as in Fig. Figure 10 and determined by the numerical integration of (7). The random walk data are generated by averaging over 50 random walk realizations.

**Figure 10:**
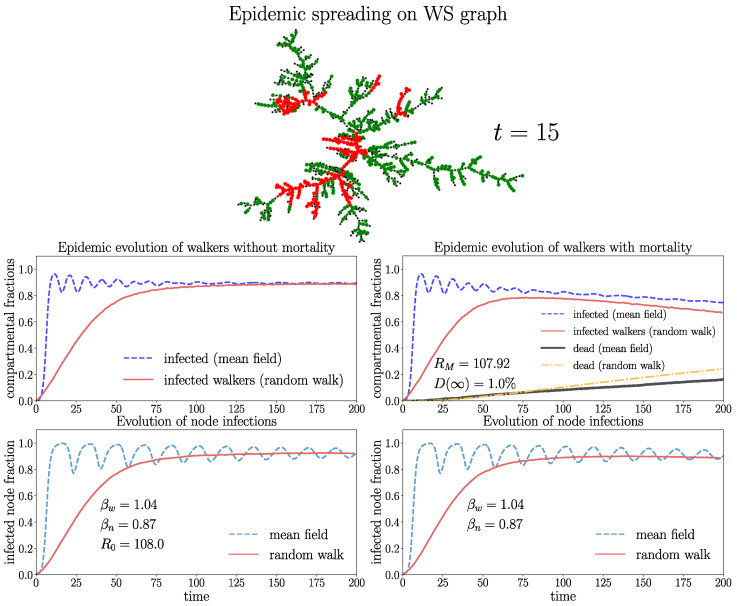
Evolution on the WS graph with Z=1000 walkers and N=1000 nodes for the same parameters as in Figure 9 averaged over 50 random walk realizations, but with reduced connectivity parameter m=〈k〉=2. The upper frame shows a snapshot (t=15) of the spreading in one random walk realization (susceptible walkers green, susceptible nodes black, infected nodes red).

Figure 9 and Figure 10 show the evolution in the WS graphs with identical parameters and Gamma distributions of $t_{I}^{w,n},t_{M}$ as in Figure 8, but with a different mortality rate parameter $\xi_{M}$ and a much smaller overall mortality D(∞)≈1%. The different network connectivity leads to different values of $\beta_{w,n}$ and mean-field solutions in Figure 9 and Figure 10. In addition, the networks of Figure 9 and Figure 10 have different connectivity features. The WS graph of Figure 9 is small-world (strongly connected), whereas the WS graph in Figure 10 is weakly connected and large-world. Comparing Figure 9 and Figure 10 shows that the network with higher connectivity of Figure 9 has higher values of $R_{0}$ or $R_{M}$, respectively. The graph in Figure 9 is small-world with larger connectivity parameter m=〈k〉=8 coinciding with the average degree, i.e., has shorter average distances between nodes as the graph of Figure 10, which has connectivity parameter m=〈k〉=2 with clearly larger average distances between the nodes. The graph of Figure 9 with higher connectivity has $R_{0}\approx 183.3$ ($R_{M}\approx 183.17$), whereas the graph of Figure 10 with lower connectivity has much smaller $R_{0}\approx 108$ ($R_{M}\approx$ 107.92). Hence, the spreading (at t=0) in the graph with higher connectivity is much stronger as in the less well-connected structure. The effect of the smaller connectivity on the spreading can also be clearly seen by means of the delayed increase of the infection numbers produced by the random walk simulations (red curves) in Figure 10. One further observes in Figure 9 that the infection numbers exhibit strong and immediate increases followed by attenuated oscillations around the endemic equilibrium (for zero mortality) with high values $J_{w}^{e}\approx 0.9$ and $J_{n}^{e}\approx 0.95$. The basic reproduction numbers with mortality are in both graphs only slightly smaller as $R_{0}$. This is due to a rather small overall mortality of D(∞)≈0.01. This effect can also be seen in the small overlap of the Gamma distributions of $t_{I}^{w}$ and $t_{M}$ in the histogram. Recall that a small value of D(∞) does not necessarily mean small $d_{w}\left(\infty \right)$ since this quantity depends also on the infection rates and network topology (see (17)) and is sensitive to repeated infections, which may indeed play an important role here as $\langle t_{I}^{w}\rangle =8$ is rather small.

In Figure 10, the infections of the random walk simulations are increasing more slowly (red curves) compared to Figure 9. The structure with higher connectivity in Figure 9 shows excellent quantitative agreement of the random walk and mean-field solutions for the walkers and nodes, well capturing the attenuated oscillations, especially for zero mortality. In the network with smaller connectivity of Figure 10, the increase of the infections is delayed compared to the mean field. On the other hand, for non-zero mortality, the mean-field and random walk dynamics for the walkers diverge slightly with time. We infer that mortality may deviate the infection rates from (1).

The comparison of the spreading in Figure 9 and Figure 10 shows clearly the role of the connectivity: the mean-field model better captures the spreading in networks with higher connectivity (short average distances between nodes) and with low mortality. The following cases give further evidence for these observations.

**Figure 11:**
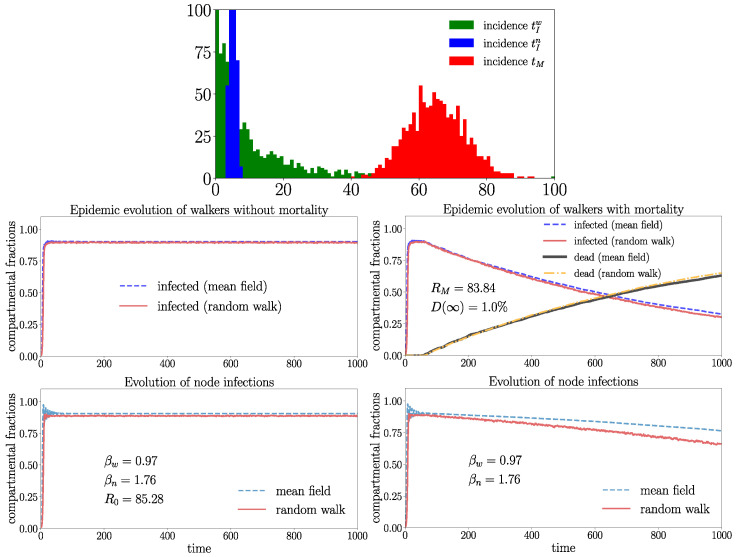
Evolution on the ER graph (nx.erdos_renyi_graph(N=1000,p=0.1)) with Z=1000 walkers and small probability p=0.1 (above the percolation limit $p_{c}=0.01$ to ensure a connected structure). The parameters are $\langle t_{I}^{n}\rangle =5$, $\xi_{I}^{n}=10$, $\langle t_{I}^{w}\rangle =10$, $\xi_{I}^{w}=0.05$, $\langle t_{M}\rangle =65$, $\xi_{M}=1$. The average degree of this ER graph is very large with 〈k〉=pN=100.

Next, we explore the spreading on an ER graph in Figure 11. The agreement of random walk simulations and the mean-field model is impressive, where this holds for both with and without mortality. One can see by means of the high average degree 〈k〉=100 and the degree distribution in Figure 7 that, for these connectivity parameters, the graph is well-connected and small-world, giving strong evidence that the mean-field approach is here capturing the spreading dynamics well.

**Figure 12:**
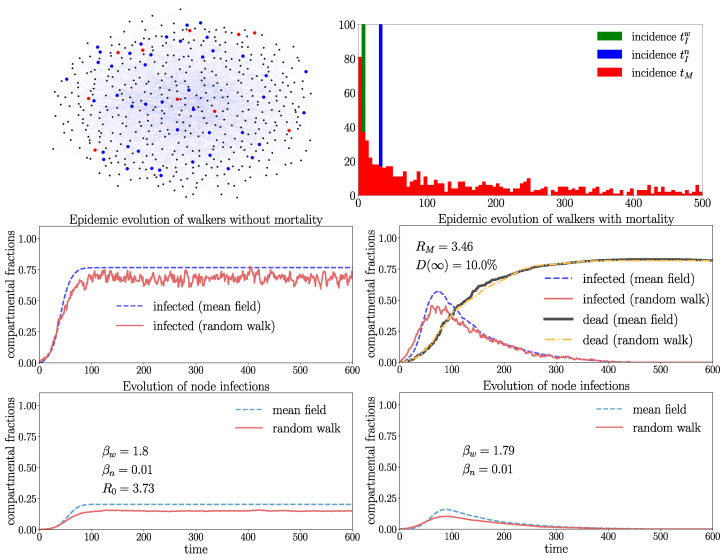
Evolution on Barabási–Albert graph with Z=50 walkers and N=5000 nodes (nx.barabasi_albert_graph(N=5000,m=5) and the average degree 〈k〉≈10) with parameters $\langle t_{I}^{n}\rangle =32$, $\xi_{I}^{n}=10^{4}$, $\langle t_{I}^{w}\rangle =8$, $\xi_{I}^{w}=10^{4}$ (sharp $t_{I}^{w,n}$), $t_{M}=500$, $\xi_{M}=10^{-3}$. The basic reproduction number $R_{M}$ is here only slightly smaller than $R_{0}$ without mortality. The left upper frame shows the initial condition. S walkers are represented in blue, I walkers in red, and nodes in black. The random walk data are generated by averaging over 10 random walk realizations.

**Figure 13:**
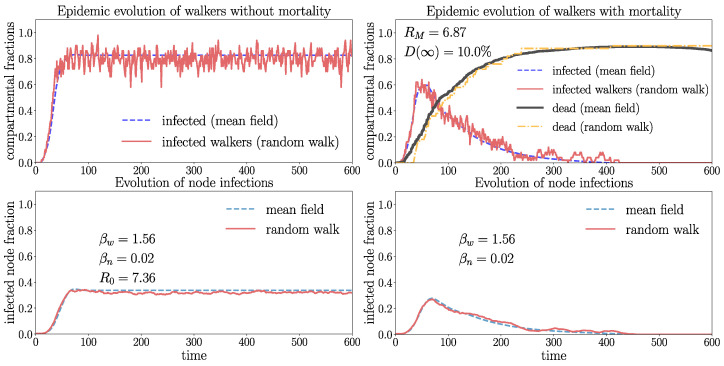
Evolution with the same parameters and number of walkers (Z=50) as in Figure 12, but fewer nodes (N=2100) for one random walk realization and average degree 〈k〉≈9.98. We interpret the increase of $R_{M}$ and $R_{0}$ due to more frequent passages of susceptible walkers on infected nodes (higher infection rates). Here, we performed only one random walk realization.

Finally, we explore in Figure 12 the dynamics on a BA network. In the right frame, we have high overall mortality of D(∞)≈10% probability for a walker to die from an infection. In this example, the disease is starting to spread as $R_{M}\approx 3.46>1$, where only a single infection wave emerges, which is made extinct by the high mortality. Recall that $R_{M}>1$ is only telling us that the healthy state is unstable, i.e., that the disease is starting to spread. It does not contain the information about whether the spreading is persistent or whether the disease is eventually made extinct. To explore the role of topological features such as the average distances between nodes, we performed the same simulation experiment with identical parameters and less (N=2100) nodes, i.e., a higher density of walkers (Figure 13).

The accordance of the mean-field model and random walk simulation is indeed also, as seen in Figure 13, excellent. We explain this by the fact that the BA network is a strongly connected structure with a pronounced small-world property. The higher density of walkers leads to increased $R_{M}$ and $R_{0}$ compared to Figure 12. There is also only a single infection wave occurring with a higher maximum value compared to Figure 12. In both cases (Figure 12 and Figure 13, right frames) the infection waves are made extinct by the high mortality of walkers, where stationary states (17) with $d_{w}\left(\infty \right)\approx 80\%$ of dead walkers are taken. When we switch off mortality (left frames), stable endemic states emerge more rapidly in Figure 13 (the case with a higher density of walkers).

Further simulation experiments (not shown here) revealed that the mean-field model and random walk simulations exhibited excellent accordance when we further increased the attachment parameters *m* or the density of walkers with otherwise identical parameters. For higher mortality, the agreement becomes less good and diverges with increasing observation time. This observation suggests that mortality modifies the infection rates in the network for larger observation times. We leave this issue for future research.

Our overall finding from this case study is that the mean-field approach (with infection rates (1)) is particularly well suited to mimic spreading in strongly connected environments with a pronounced small-world feature, but is less good for higher mortality.

## 7. Conclusions

We studied epidemic spreading in complex graphs, where we focused on the transmission pathway via vectors mimicking the spreading of a certain class of diseases such as Dengue, Malaria, Pestilence, and others. We developed a stochastic compartment model for the walkers and nodes with mortality for the walkers. For zero mortality, we obtained the endemic equilibrium in explicit form (Equation (33)). The stability analysis of the endemic and healthy states revealed the crucial control parameter for spreading, the basic reproduction number. We obtained the basic reproduction numbers $R_{M}$ and $R_{0}$ with and without mortality, respectively, where we proved that $R_{M}\leq R_{0}$ (the relations (50) with (48)). For $R_{M},R_{0}>1$, the healthy state is an unstable fixed point where the endemic equilibrium exists for zero mortality as a unique stable fixed point independent of the initial conditions. The basic reproduction numbers depend on the means of the compartmental sojourn times in compartment I of the nodes and walkers and on the topology of the network captured by the mean-field rate constants $\beta_{w,n}$. For future research, it would be desirable to extend the mean-field model in such a way that it connects the rate parameters $\beta_{w,n}$ in explicit form with the topological features of the network such as the mean distance of the nodes, average connectivity, and others. We refer to [38] for a recent study connecting the spreading of COVID-19 with such topological features, where it has to be emphasized that COVID-19 is not a vector-transmitted disease and, hence, does not refer to the class of diseases analyzed here.

Our model has applications beyond epidemic dynamics, for instance in chemical reaction models [39], and can be extended in various directions. For instance, the inclusion of additional compartments and natural birth and death processes based on real-world observations of Malaria transmission dynamics is of interest [40]. An interesting question to explore in a follow-up project is whether our class of compartment models with indirect transmission pathways may exhibit (for zero mortality) persistent oscillations (Hopf instabilities). (See our brief discussion at the end of Appendix A.2.) An open problem also remains how a large-world network topology may be included in such a mean-field model, modifying the infection rates. Further promising directions include an accounting of immune and incubation compartments, the effects of mitigation measures, and vaccinations, among many others.

## Data Availability

No new data were created or analyzed in this study. Data sharing is not applicable to this article.

## Appendix A

### Appendix A.1. Some Basic Notions

Here, we recall briefly some basic notions used in the paper. The infection rates are causal functions:

(A1) A(t)=A(t)Θ(t)

where Θ(t) indicates the Heaviside step function defined by

(A2) $$\Theta \left(\zeta \right)=\left\{\begin{array}{c} 1,\;\zeta \geq 0\\ 0,\;\zeta <0 \end{array}\right.$$

with Θ(ζ)+Θ(−ζ)=1, and in our definition, Θ(0)=1. Its derivative yields the Dirac δ-distribution $\frac{d}{d\zeta }\Theta \left(\zeta \right)=\delta \left(\zeta \right)$. We use throughout the paper mutually independent strictly positive random variables $T_{1},\ldots ,T_{n}\in R_{+}$ The random variables $T_{j}$ are assumed to follow their specific PDFs:

(A3) $$Prob\left[T_{j}\in \left[u,u+du\right]\right]=\langle \;\delta (u-T_{j})\;\rangle =K_{j}\left(u\right)du$$

where the PDFs $K_{j}$ are causal functions as a consequence of the positiveness of the $T_{j}$. Then, the averaging rule applies:

(A4) $$\langle \;f(t;T_{1},T_{2},\ldots ,T_{n})\;\rangle =\int_{0}^{\infty }\ldots \int_{0}^{\infty }dt_{1}\ldots dt_{n}f\left(t;t_{1},t_{2},\ldots ,t_{n}\right)K_{1}\left(t_{1}\right)\ldots K_{n}\left(t_{n}\right)$$

for suitable functions *f*. For $f\left(t;T_{1},T_{2},\ldots ,T_{n}\right)=g_{1}\left(T_{1}\right)\ldots g_{n}\left(T_{n}\right)$, using the independence of the $T_{j}$, this yields

$$\langle \;g_{1}\left(T_{1}\right)\ldots g_{n}\left(T_{n}\right)\;\rangle =\langle \;g_{1}\left(T_{1}\right),\rangle \ldots \langle \;g_{n}\left(T_{n}\right)\;\rangle .$$

Important cases emerge by applying (A4) to the exponentials:

(A5) $$\langle \;e^{-\lambda (T_{1}+\ldots +T_{n})}\;\rangle =\int_{0}^{\infty }e^{-\lambda t}\langle \delta (t-T_{1}-\ldots -T_{n})\rangle dt={\hat{K}}_{1}\left(\lambda \right)\ldots {\hat{K}}_{n}\left(\lambda \right),\;ℜ\left\{\lambda \right\}\geq 0$$

In this relation, the LTs of the PDFs come into play:

(A6) $${\hat{K}}_{j}\left(\lambda \right)=\int_{0}^{\infty }e^{-\lambda t}K_{j}\left(t\right)dt$$

where ${\hat{K}}_{j}\left(\lambda \right){|}_{\lambda =0}=1$ reflects the normalization of PDFs (A3). A further observation is

(A7) $$\langle \delta (t-T_{1}-\ldots -T_{n})\rangle =\left(K_{1}★\ldots ★K_{n}\right)\left(t\right)$$

where *★* stands for the convolution $\left(K_{1}★K_{2}\right)\left(t\right)=\int_{0}^{t}K_{1}\left(\tau \right)K_{2}\left(t-\tau \right)d\tau$ of the causal PDFs.

### Appendix A.2. Proof of Stability of the Endemic Equilibrium

Here, we develop the rest of the proof of the stability of the endemic equilibrium, i.e., we show that the function (42) is strictly positive for μ≥0 with $R_{0}-1>0$. First, we observe that ${\hat{\Phi }}_{I}^{w,n}\left(\mu \right)\leq \langle t_{I}^{w,n}\rangle$ with ${\hat{\Phi }}_{I}^{w,n}\left(0\right)=\langle t_{I}^{w,n}\rangle$ and ${\hat{\Phi }}_{I}^{w,n}\left(\infty \right)=0$. For our convenience, we introduce the functions

(A8) $$\lambda_{w,n}\left(\mu \right)=\frac{{\hat{\Phi }}_{I}^{w,n}\left(\mu \right)}{\langle t_{I}^{w,n}\rangle }\in \left(0,1\right]$$

which are the LTs of the normalized functions $\Phi_{I}^{w,n}\left(t\right)/\langle t_{I}^{w,n}\rangle$ and which are, by virtue of Bernstein’s theorem [41], completely monotonic (CM) with respect to μ, i.e.,

(A9) $${\left(-1\right)}^{n}\frac{d^{n}}{d\mu^{n}}\lambda_{w,n}\left(\mu \right)\geq 0,\;\mu \in \left(0,\infty \right)$$

inheriting this feature from the exponential $e^{-\mu \tau }$ (t>0). Therefore,

$$\frac{d}{d\mu }\lambda_{w,n}\left(\mu \right)=-\frac{1}{\langle t_{I}^{w,n}\rangle }\int_{0}^{\infty }e^{-\mu t}t\Phi_{I}^{w,n}\left(t\right)dt<0$$

(as $\Phi_{I}^{n,w}\left(t\right)\in \left(0,1\right]$) exists; thus, $\lambda_{w,n}\left(\mu \right)$ is monotonously decreasing with μ with $\lambda_{w,n}\left(0\right)=1\geq \lambda_{w,n}\left(\mu \right)>0$. Further, we observe in Equation (33) that $J_{n}^{e}\beta_{w}\langle t_{I}^{w}\rangle =\frac{R_{0}-1}{1+\beta_{n}\langle t_{I}^{n}\rangle }$, $J_{w}^{e}\beta_{n}\langle t_{I}^{n}\rangle =\frac{R_{0}-1}{1+\beta_{w}\langle t_{I}^{w}\rangle }$; thus,

$$R_{0}\left(J_{n}^{e}+J_{w}^{e}\right)=\left(R_{0}-1\right)\left(\frac{\beta_{n}\langle t_{I}^{n}\rangle }{1+\beta_{n}\langle t_{I}^{n}\rangle }+\frac{\beta_{w}\langle t_{I}^{w}\rangle }{1+\beta_{w}\langle t_{I}^{w}\rangle }\right).$$

Then, $G_{e}\left(\mu \right)$ reads

(A10) $$\begin{array}{ccc} G_{e}\left(\mu \right) & =1+\lambda_{w}\left(\mu \right)\lambda_{n}\left(\mu \right)\left\{-R_{0}+\left(R_{0}-1\right)\left(\frac{\beta_{n}\langle t_{I}^{n}\rangle }{1+\beta_{n}\langle t_{I}^{n}\rangle }+\frac{\beta_{w}\langle t_{I}^{w}\rangle }{1+\beta_{w}\langle t_{I}^{w}\rangle }\right)\right\} & \\ & \;+\left(R_{0}-1\right)\left(\frac{\lambda_{w}\left(\mu \right)}{1+\beta_{n}\langle t_{I}^{n}\rangle }+\frac{\lambda_{n}\left(\mu \right)}{1+\beta_{w}\langle t_{I}^{w}\rangle }\right). \end{array}$$

Now, we observe that $\lambda_{w}\left(\mu \right)\lambda_{n}\left(\mu \right)\leq \lambda_{w,n}\left(\mu \right)$; thus, a lower bound function $H\left(\mu \right)\leq G_{e}\left(\mu \right)$ is generated by replacing $\lambda_{w,n}\left(\mu \right)\to \lambda_{w}\left(\mu \right)\lambda_{n}\left(\mu \right)$ in the second line. Now, it is sufficient to prove that 0<H(μ). We, hence, obtain for this lower bound function:

(A11) $$\begin{array}{ccc} H(\mu ) & =1+\lambda_{w}\left(\mu \right)\lambda_{n}\left(\mu \right)\left\{-R_{0}+\left(R_{0}-1\right)\left(\frac{\beta_{n}\langle t_{I}^{n}\rangle }{1+\beta_{n}\langle t_{I}^{n}\rangle }+\frac{\beta_{w}\langle t_{I}^{w}\rangle }{1+\beta_{w}\langle t_{I}^{w}\rangle }\right)+\left(R_{0}-1\right)\left(\frac{1}{1+\beta_{n}\langle t_{I}^{n}\rangle }+\frac{1}{1+\beta_{w}\langle t_{I}^{w}\rangle }\right)\right\} & \\ \; & =1+\lambda_{w}\left(\mu \right)\lambda_{n}\left(\mu \right)\left(R_{0}-2\right) & \\ \; & =1-\lambda_{w}\left(\mu \right)\lambda_{n}\left(\mu \right)+\left(R_{0}-1\right)\lambda_{w}\left(\mu \right)\lambda_{n}\left(\mu \right) \end{array}$$

and with $1-\lambda_{w}\left(\mu \right)\lambda_{n}\left(\mu \right)\geq 0$ and $\left(R_{0}-1\right)\lambda_{w}\left(\mu \right)\lambda_{n}\left(\mu \right)>0$, it follows that $0<H\left(\mu \right)\leq G_{e}\left(\mu \right)$, concluding the proof of the stability of the endemic equilibrium.

### Appendix A.3. A Few Remarks on the Possibility of Oscillatory (Hopf) Instabilities of the Endemic Equilibrium

Let us briefly explore whether an oscillatory (Hopf) instability of the endemic equilibrium is possible. To that end, we write $G_{e}\left(\mu \right)$ as

(A12) $$G_{e}\left(\mu \right)=1+\sigma \hat{g}\left(\mu \right)\geq 1-\hat{g}\left(\mu \right)$$

where σ=±1 and $\hat{g}\left(\mu \right)$ is a non-negative CM function (see Figure 4) with the maximum value $\hat{g}\left(0\right)=\left|R_{0}-2\right|$. Then, the following two cases may occur.

*Case (i)* σ=−1; $0<G_{e}\left(0\right)=R_{0}-1<1$ ($1<R_{0}<2$):

Then, $\hat{g}\left(\mu \right)$ can be represented as the LT of a non-negative function g(t), and consider, now, $\mu =\mu_{1}+i\mu_{2}$ with $\mu_{1}\geq 0$; thus, the real part of $\hat{g}\left(\mu_{1}+i\mu_{2}\right)$ can be written as

(A13) $$ℜg\left(\mu_{1}+i\mu_{2}\right)=\int_{0}^{\infty }g\left(t\right)e^{-\mu_{1}t}\cos\left(\mu_{2}t\right)dt,\;\mu_{1}\geq 0$$

with $\hat{g}\left(0\right)=2-R_{0}$ and, clearly, $-\hat{g}\left(\mu_{1}\right)\leq ℜg\left(\mu_{1}+i\mu_{2}\right)\leq \hat{g}\left(\mu_{1}\right)$. Therefore,

(A14) $$ℜG_{e}\left(\mu_{1}+i\mu_{2}\right)=1-ℜg\left(\mu_{1}+i\mu_{2}\right)\geq 1-\hat{g}\left(\mu_{1}\right)\geq 1-\hat{g}\left(0\right)=R_{0}-1>0.$$

Hence, in the range of case (i) $1<R_{0}<2$, there is no oscillatory (Hopf) instability of the endemic state possible (a similar consideration of function G(μ) of (39) shows as well that the healthy state for $R_{0}<1$ does not exhibit an oscillatory instability):

*Case (ii)* σ=+1; $R_{0}-1>1$:

Here, we have two pertinent ranges of $R_{0}$. The first is the range (a) $\hat{g}\left(0\right)=R_{0}-2<1$ (i.e., $R_{0}<3$), and the second one (b) is $\hat{g}\left(0\right)=R_{0}-2>1$. Clearly, in the range (a), (A14) remains true and $ℜG_{e}\left(\mu_{1}+i\mu_{2}\right)$ strictly positive. Hence, for $1<R_{0}<3$, no Hopf instability is possible.

This changes in the range (b), since $\hat{g}\left(0\right)=R_{0}-2>1$; thus, $ℜG_{e}\left(\mu_{1}+i\mu_{2}\right)=1+ℜg\left(\mu_{1}+i\mu_{2}\right)$ may become negative. Therefore, for $R_{0}>3$, a Hopf instability of the endemic state becomes *possible*. However, the possibility that $ℜG_{e}\left(\mu_{1}+i\mu_{2}\right)=0$ is only necessary, but not sufficient for a Hopf instability. One also needs simultaneously that the imaginary part $ℑG_{e}\left(\mu_{1}+i\mu_{2}\right)=0$ is vanishing for the same $\mu =\mu_{1}+i\mu_{2}$. In the simulations performed for this paper, we did not observe persistent oscillations. We leave the exploration of this issue for future research in a follow-up project.

### Appendix A.4. A Very Brief Recap of Random Graphs

Here, we recall briefly some essential features of the three classes of random graphs, which we use in the random walk simulations. For an extended outline, consult, e.g., [42]. The three classes of random graph models depicted hereafter are motivated by the observation that complex random network structures are encountered ubiquitously and crucially determine human and animal mobility patterns, including epidemic propagation.

(i) Erdös and Rényi (ER) graph

The ER graph is one of the most basic variants of a random graph, which was introduced in 1959 by Erdös and Rényi [43]. We use the so-called G(N,p) variant of the random ER graph model, which is actually due to Gilbert [44], and generated as follows [11,14]. Given are *N* labeled nodes. Any pair of nodes is connected independently by an edge with uniform probability *p*. The probability $P_{N}\left(k\right)$ that a node has 0≤k≤N−1 connections is given by a binomial distribution:

$$P_{N}\left(k\right)=\left(\begin{array}{c} N-1\\ k \end{array}\right)p^{k}{\left(1-p\right)}^{N-1-k}\to \frac{{\langle k\rangle }^{k}}{k!}e^{-\langle k\rangle }$$

where 〈k〉=(N−1)p∼Np denotes the average degree. For N→∞ (while Np is kept constant), the degree distribution $P_{N}\left(k\right)$ converges to a Poisson distribution representing the infinite graph limit of the ER G(N,p) model. Therefore, $P_{N}\left(k\right)$ is rapidly decaying with degree *k*, so the number of nodes with a high number of connections is very small. In order to obtain in the G(N,p) model a connected graph, it is necessary that $p>p_{c}=\frac{logN}{N}$ be above the percolation limit [30,43]:

(ii) Watts–Strogatz (WS) network:

The WS graph model [11,45,46] starts with a ring of *N* nodes, where each node is connected symmetrically with a number m<<N to the left and right neighbor nodes by an edge such that each node has 2m connections. In the second step, each of the connections i,j of node *i* is replaced with probability *p* by a randomly chosen connection i,k uniformly among other nodes avoiding self-connections and link duplication, so that each connection i,k is chosen only once. There are two noteworthy limits for a WS graph. For p=0 (no rewiring of links), we have a regular ring with constant degree 2m for all nodes. In the limit p=1, an ER graph is emerging with probability 2m/(N−1) for a link. The WS graph has the small-world property (short average distances between pairs of nodes) and a high tendency to develop clusters of nodes [46].

(iii) Barabási–Albert (BA) graph:

The BA graph is generated by a preferential attachment mechanism for newly added nodes [14,15,42,47]. One starts with $m_{0}$ nodes and adds new nodes. Any newly added node is connected with $m\leq m_{0}$ existing nodes (*m* is referred to as the attachment parameter), most likely with nodes of high degrees. In this way, nodes with a high degree receive further links. This leads to an asymptotically scale-free network with a power law degree distribution:

$$P\left(k\right)\propto k^{-2-\mu },\;\mu \approx 1.$$

As the decrease in this power law is relatively slow, there might exist quite a few nodes with many links (hub nodes) and many nodes with few links. BA graphs are believed to mimic a large class of real-world networks including the World Wide Web and citation, social, and metabolic networks.

Realizations of these three types of random graphs are used in our multiple random walker simulations and shown in Figure 7.
